# Extending Without Extensors: Morphofunctional Insights and Qualitative Experiments on Myriapod Legs

**DOI:** 10.1002/jmor.70171

**Published:** 2026-09-23

**Authors:** Birk Rillich, Benjamin Naumann, Tom Weihmann, Christian S. Wirkner

**Affiliations:** ^1^ Allgemeine & Spezielle Zoologie Institut für Biowissenschaften, Universitat Rostock Rostock Germany; ^2^ Institut für Biowissenschaften, Tierphysiologie Universitat Rostock Rostock Germany

**Keywords:** Arthropoda, functional morphology, leg joints, muscles, Myriapoda, zoology

## Abstract

Until recently, the absence of intrinsic extensor muscles in the distal leg joints of Myriapoda—a condition previously recognized in spiders—was known but scattered across various publications, lacking a consolidated overview. This has led to various hypotheses regarding the mechanisms underlying the extension of these so‐called hinge joints. Hemolymph pressure has been proposed as a possible explanation, along with the pressing of the leg tip against the ground and the elasticity of the joints. The aim of this study is to investigate the morpho‐functional background responsible for the extension of the joints and whether the absence of intrinsic extensor muscles was a feature present in the ground pattern of the Myriapoda. To this end, we examined the cuticle and muscle anatomy of representatives from the four major taxa—Chilopoda, Symphyla, Pauropoda, and Diplopoda—using confocal laser scanning microscopy, micro‐computed tomography, and semi‐thin sections. Additionally, extension experiments have been conducted on the legs of freshly euthanized *Spirostreptus* spec. 1 “Tanzania” (Diplopoda) and *Lithobius forficatus* (Chilopoda). All intrinsic and extrinsic muscles of the investigated species were described, along with the cuticle anatomy of the joints and their autofluorescent properties. The findings confirm that hinge joints lacking intrinsic extensor muscles were likely already present in the ground pattern of Myriapoda. Furthermore, three main extension mechanisms of the hinge joints are hypothesized. These mechanisms are: (i) the generation of hemolymph pressure within the leg, (ii) elasticity of the joints' cuticular structures, and (iii) pressing the tip of the leg against the ground.

## Graphical abstract

The distal joints of myriapods are hinge joints and therefore cannot be extended by intrinsic muscles. Using confocal laser scanning microscopy, µCT, semi‐thin sections, and experimental approaches, we investigated the morphology and extension mechanisms of myriapod legs. Our results indicate three contributing mechanisms: contraction of extrinsic retractors and intrinsic depressors, hydraulic pressure, and joint elasticity.

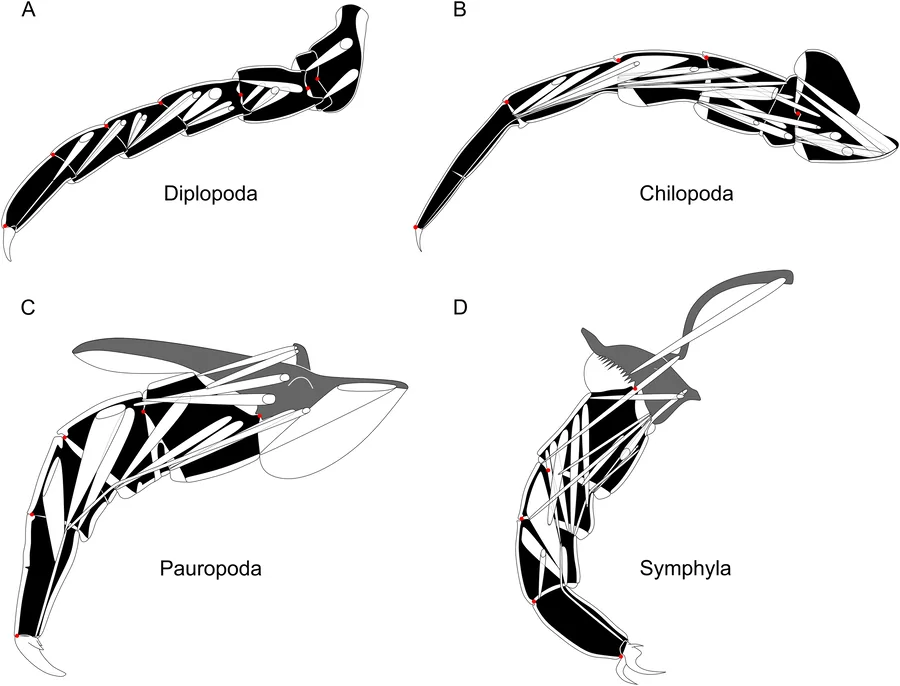

## 1 Introduction

**Figure 1:**
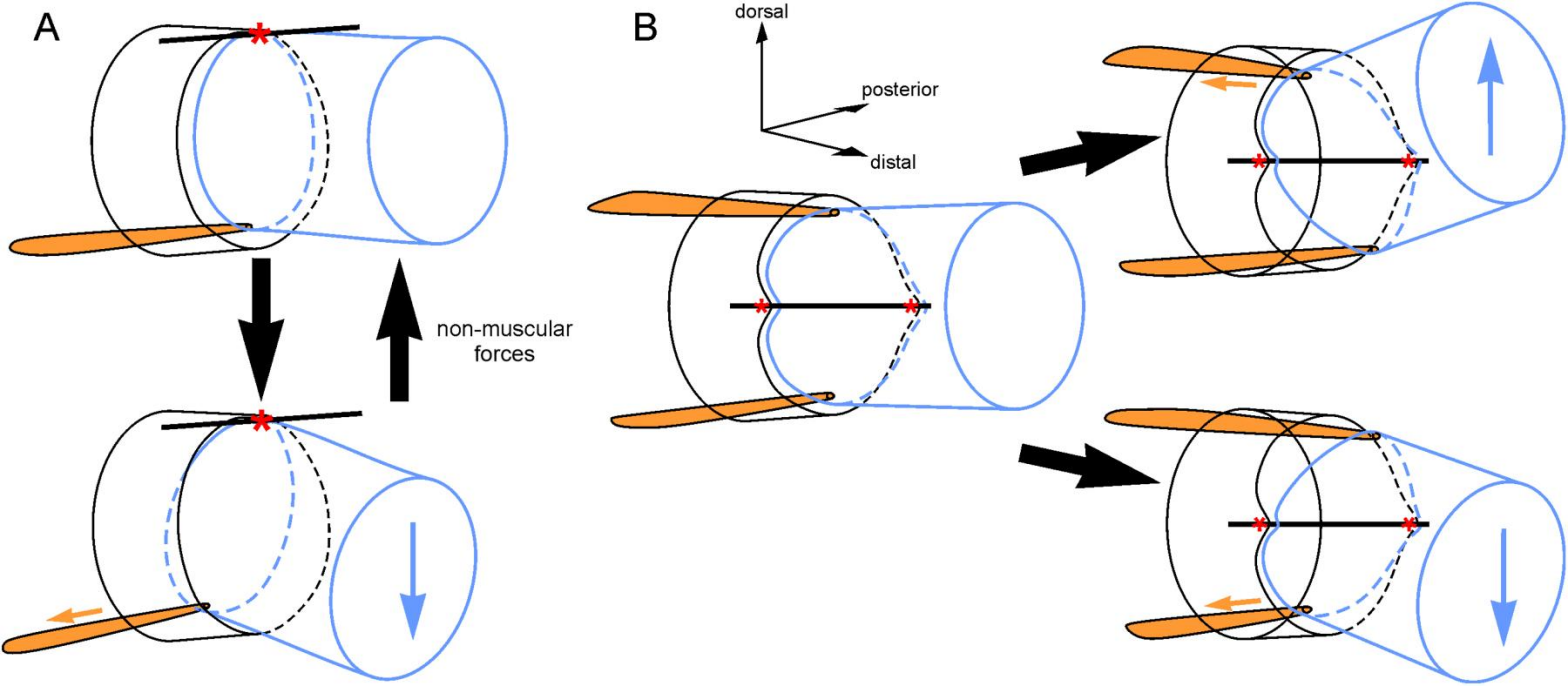
Schematic drawings of (A) a hinge joint and (B) a dicondylic pivot joint. Because the axis of rotation is positioned tangentially, the hinge joint cannot be extended by muscular force and allows only flexion (e.g., Manton 1954, 1957, 1958a, 1958b). In contrast, both muscular extension and flexion are possible in a dicondylic pivot joint.

Myriapoda are one of the four major groups of Arthropoda and include Chilopoda, Symphyla, Pauropoda, and Diplopoda (Edgecombe 2004; Benavides et al. 2023). Their bodies consist of a head followed by a variable number of leg‐bearing segments. As with other Arthropods, myriapod legs are composed of sclerotized, cylindrical podomeres that are movably connected by arthrodial membranes. In most arthropod joints, the rotational axis between two podomeres is determined by two opposing joint heads that engage in the joint sockets of the adjacent podomere, forming a dicondylic pivot joint (Snodgrass 1958; Manton 1958a, 1977). The movement of the distal podomere relative to the proximal one is achieved through the action of antagonistic muscles (Figure 1B).

The walking legs of all investigated Myriapoda have one thing in common: the distal joints lack intrinsic extensor muscles from the trochanter–prefemur joint or the joint immediately distal to it (Figure 1A, after Weihmann et al. 2010; Manton 1958a; and others). Although Tiegs (1940) initially described such muscles in Symphyla, Manton (1966) later reinterpreted them as protractors of the tarsus, reinforcing the broader observation. The distal joints of myriapod walking legs are hinge joints, as it has been shown for Chilopoda (Manton 1965; Rilling 1968), Pauropoda (Ewing 1928; Manton 1966), Symphyla (Ewing 1928; Manton 1966), and Diplopoda (Manton 1954, 1957, 1958b, 1961). Hinge joints are characterized by dorsal articulation, meaning that the pivot point is located dorsally on the joint (Manton 1958b). Consequently, no muscle can pull dorsally to the rotational axis, which makes intrinsic muscular extension of the hinge joints impossible (Ewing 1928; Tiegs 1947; Manton 1954, 1957, 1958a, 1958b, 1961, 1965, 1966; Snodgrass 1958). A similar situation exists in the legs of chelicerates (except Pantopoda [Manton 1977]), which lack intrinsic extensor muscles in certain leg joints—a phenomenon that has caused significant scientific interest over time (Manton 1958a, 1973; Parry and Brown 1959; Wilson 1970; Anderson and Prestwich 1975; Weihmann et al. 2012; Runge and Wirkner 2019).

The peculiar lack of intrinsic extensor muscles raises the question of how myriapod legs extend. Several alternative hypotheses have been put forward to answer this question. One suggests that muscle‐induced pressing of the leg tip against the substrate during stance leads to its extension (Manton 1958a, 1965). The step cycle of myriapods can be divided into two phases: (1) the propulsive backstroke, during which the tip of the leg contacts the substrate and the leg moves from anterior to posterior, and (2) the recovery stroke, in which the leg tip is off the substrate and moves from posterior to anterior (Manton 1952, 1954, 1965, 1966). The antero–posterior movement originates almost entirely from the trunk–coxa joint (Manton 1965, 1966). In the first half of the backstroke, the leg also bends due to intrinsic flexors until it has completed half of the propulsive backstroke. From the middle of the backstroke onwards, the leg extends, with the dorsal side of the leg being rotated forward by the trunk–coxa joint (Manton 1965, 1966). Thereby, the extrinsic retractor and intrinsic proximal depressor muscle press the tip of the leg against the substrate (Manton 1965). But how does this work in detail, and can propulsion be generated in the process?

Another hypothesis focuses on the (additional) role of hemolymph pressure. Since complete extension occurs during the recovery stroke, when the leg tip is lifted off the substrate (Manton 1958a, 1958b), it seems clear that other mechanisms must also be involved in the extension process (Manton 1958a). Hemolymph pressure necessary to extend the hinge joints cannot be generated by the heart, as the generated pressure is 30 to 100 times too low (Manton 1958a). Therefore, additional muscles may increase hemolymph pressure locally (Manton 1958a), or the required pressure may be generated within the trunk. Additionally, unlike the legs of scorpions (Alexander 1958, 1967) and Solifugae (Sensenig and Shultz 2004), no elastic sclerites are known in the joints of Myriapoda (Manton 1958a), which would enable passive extension through joint elasticity.

An important component of arthropod legs that contributes to arthropodial movement and that has not been investigated in Myriapoda is resilin. Resilin, an elastic, rubber‐like protein first described in detail by Weis‐Fogh (1960), is found in various locations in the cuticle of many arthropods and is, for example, assumed to be primarily responsible for the elasticity of the non‐muscular extensible patella–tibia and tibia–tarsus joints of Solifugae (Shultz 1989; Sensenig and Shultz 2004). With the development of improved detection methods, such as confocal laser scanning microscopy (cLSM), it has become significantly easier to detect resilin compared to the mid‐20th century (Michels and Gorb 2012). To better understand the functional morphology of myriapod walking legs and answer the aforementioned question, we investigate the cuticle and muscle anatomy of representatives of the four major myriapod taxa. In addition, extension experiments were carried out with freshly euthanized *Lithobius forficatus* (Linnaeus, 1758) (Chilopoda) and *Spirostreptus* spec. 1 “Tanzania” (Diplopoda).

Beyond investigating the forces and mechanisms driving hinge‐joint extension, our morpho‐functional analyses provide fine‐scale anatomical information on podomere arrangement, articulation, and muscular connections. Such data are essential for evaluating hypotheses of podomere homology, which until now have relied largely on morphology and yielded conflicting results (e.g., Manton 1965; Rilling 1968). Although gene‐expression approaches have informed homology assessments in other arthropods (Bruce and Patel 2020), comparable analyses are lacking for myriapods. Our findings, therefore, offer an extended anatomical framework that may help test or refine existing concepts of myriapod limb homology.

## 2 Material and Methods

### 2.1 Taxa Sampling

The data on the collection of the animals used in this study are listed in Table 1. During taxon sampling, care was taken to represent as many major taxa of Myriapoda as possible.

**Table 1:** Collection data of the animals used in this study.

Species		Collector	Location	Special sampling techniques	Fixative	Number of investigated individuals
*Scutigera coleoptrata* (Linnaeus, 1758)	Chilopoda, Scutigeromorpha	Carsten H. G. Müller	Island of Ibiza, Spain	—	70% Ethanol, Dubosq‐Brasil	3
*Lithobius forcifatus* (Linnaeus, 1758)	Chilopoda Lithobiomorpha	Birk Rillich	Germany, Rostock, 54°04′49.9″ N 12°05′57.5″ E	—	70% Ethanol, Dubosq‐Brasil	10
*Stigmatogaster subterranea* (Shaw, 1789)	Chilopoda, Geophilomorpha	Birk Rillich	Germany, Rostock, 54°04′49.9″ N 12°05′57.5″ E	—	70% Ethanol, 4% PFA	4
*Scolopendrellopsis subnuda* (Hansen, 1903)	Symphyla, Scolopendrellidae	Birk Rillich	Germany, Rostock, 54°10′28.1″ N 12°10′22.7″ E	Berlese funnel	70% Ethanol	4
*Scutigerella immaculata* Newport, 1845	Symphyla, Scutigerellidae	Nikolaus Szucsich	Austria, Prellenkirchen and Eschenau	—	Dubosq‐Brasil	1
*Acopauropus ornatus* (Latzel, 1884)	Pauropoda, Eurypauropodidae	Klaus Hasenhütl	Slovenia, 47°11′24″ N; 15°35′23″ E	sieving	70% Ethanol	3
*Polyxenus lagurus* (Linnaeus, 1758)	Diplopoda, Penicillata	Birk Rillich	Germany, Rostock, 54°11′07.7″ N 12°10′40.2″ E	entomological aspirator	70% Ethanol, 4% Formalin in 1× PBS	20
*Spirostreptus* spec. 1 “Tanzania”	Diplopoda, Spirostreptida	Benjamin Naumann	From cultivation of the Allgemeine & Spezielle Zoologie, Rostock	—	70% Ethanol	5
*Benoitolus siamensis* (Attems 1936)	Diplopoda, Spirobolida	Benjamin Naumann	From cultivation of the Allgemeine & Spezielle Zoologie, Rostock	—	70% Ethanol	1

### 2.2 Fixatives

Depending on the technique to be applied, three different fixatives were used. The samples examined with the cLSM were fixed with 4% bPFA (4% Paraformaldehyde in 1× PBS) or 70% ethanol. The animals scanned with micro‐computed tomography (µCT) were fixed with Dubosq Brasil (one part picric acid to 60 parts formalin (40%), 15 parts glacial acetic acid, and 150 parts ethyl alcohol (80%) [Rillich and Oliveira 2023]) or 4% bPFA. For a detailed breakdown of which samples were fixed and how they were further processed, see Appendix S1: Tables 2 and 3.

### 2.3 cLSM

A Leica Stellaris 8 cLSM was used to examine the cuticle and musculature of all investigated species. Samples were mounted using the “sandwich” method of Barbosa et al. (2014), positioned between two cover slips separated by small pieces of modeling clay to avoid compression. To enhance transparency and laser penetration, specimens were cleared and mounted in BABB (1:2 benzyl alcohol:benzyl benzoate), Rapiclear 1.47 (SunJin Lab), or Hoyer's medium (Anderson 1954), following Chetverikov (2012). For each species, one specimen was additionally mounted in 100% glycerin to assess whether clearing affected cuticular autofluorescence. Treatment details for individual specimens are listed in Appendix S1: Table 2.

Arthropod cuticle consists of multiple layers with distinct physical and optical properties (Vincent 2002; Andersen 2003). Several components exhibit natural autofluorescence (Klaus et al. 2003; Klaus and Schawaroch 2006; Michels 2007) and can be selectively excited by specific laser wavelengths (Michels and Gorb 2012; Michels et al. 2012), enabling visualization of both structure and material composition. Three material‐related signals were distinguished:

– Potentially Resilin‐rich regions: Highly elastic areas containing large amounts of resilin are specifically excited at 405 nm (Michels and Gorb 2012).
– Flexible cuticle: These softer, yet stable regions are excited at 488 and 555 nm (Michels and Gorb 2012).
– Sclerotized cuticle: These rigid structures primarily respond to excitation at 633 nm (Michels and Gorb 2012).

The cLSM detectors record emitted photons as grayscale values; colors were assigned during processing according to excitation wavelength (blue = 405 nm, yellow = 488 nm, red = 633 nm), unless stated otherwise. While cLSM provides qualitative information on cuticle composition, it does not allow quantitative material analysis. For methodological considerations and limitations, see Josten et al. (2022). The protocol used here indicates the presence of resilin but does not confirm it; definitive verification requires pH‐dependent autofluorescence (Neff et al. 2000) or antibody labeling (Burrows et al. 2011).

### 2.4 µCT

In order to study and visualize the internal anatomy of the animals, the body parts of interest were scanned using µCT. To enhance the contrast of the musculature in the µCT scans, the specimens were either submerged in 3% phosphotungstic acid (PTA) in 90% ethanol and incubated for several days in a water bath heated to 40°C, or submerged in 1% iodine in ethanol at room temperature for several days. Specimens treated with PTA were scanned in 90% ethanol, while those treated with iodine were transferred to 99.8% ethanol and then critical‐point dried using a Leica EM CPD300 before scanning. For a detailed protocol of the treatment of individual samples, see Appendix S1: Table 3. The samples were then scanned using an X‐ray microscope, Zeiss XRadia Versa 410. *Benoitolus siamensis* (Attems 1936) was scanned at Deutsches Elektronen‐Synchrotron DESY of the Helmholtz using propagation‐based phase‐contrast tomography (Hehn et al. 2018).

All datasets can be acquired by request from the corresponding author(s).

### 2.5 Semi‐Thin Sections

One individual each of *Acopauropus ornatus* (Latzel 1884) and *Scolopendrellopsis subnuda* (Hansen 1903) was sectioned. The semi‐thin sections were cut as described in Wirkner and Richter (2009). Furthermore, a transversal semi‐thin section series (1 µm thickness) of *Scutigerella immaculata* Newport (1845), kindly provided by Nikolaus Szucsich, Vienna, was also analyzed. To digitalize the sections, an Axioscan 7 slide scanner (Zeiss) was used.

### 2.6 3D Reconstructions and 3D Implementation

The digital image stacks obtained by μCT, cLSM, and digitalized semi‐thin sections were processed with the 3D reconstruction software Amira 2021.2 (Thermo Fisher Scientific). Based on the segmentation, it is possible to create a surface rendering and Volrens to visualize the morphology. These 3D surfaces were then further processed with the software Meshlab 2020.12 (Open‐Source), then exported as a PDF with Adobe Acrobat 9 Pro Extended, and implemented in the existing PDF of this study. This allows all readers to open the 3D models with one click to gain a better insight into the anatomy and spatial relationships between morphological features of the animals (Rillich and Wirkner 2025).

### 2.7 Preparation of Figure Plates

Figure plates were created using Corel Graphics Suite 2017. The preparation and imaging of the cLSM scans were performed using Leica Application Suite X (Leica) and Amira 2021.2 (Thermo Fisher Scientific).

### 2.8 Muscle Terminology

Muscle terminology follows Runge and Wirkner (2019). Muscles were treated as functional units, meaning that groups of fibers with closely adjacent insertion sites were classified as a single muscle. Naming is based on the proximal and distal insertion points relative to the cuticle. Each muscle name consists of three elements: (1) the abbreviation of the proximal insertion structure, (2) a dash, and (3) the abbreviation of the distal insertion structure, followed by a Roman numeral indicating the muscle's rank.

If multiple structures of the same type serve as attachment sites (e.g., several tendons or muscle nodes), an Arabic numeral is added to the abbreviation to ensure unambiguous identification. All muscles connecting a given proximal structure to a given distal structure are numbered consecutively according to the anterior–posterior position of their proximal insertion. More anterior insertions receive a higher rank (lower Roman numeral). If two muscles insert equally anteriorly, the more proximal one ranks higher; if both are equally anterior and proximal, the more dorsal muscle receives the higher rank.

For example, Fe‐Ta1I designates a muscle inserting proximally on the femur and distally on tarsus 1, with the highest rank among all muscles connecting these two structures.

**Figure 2:**
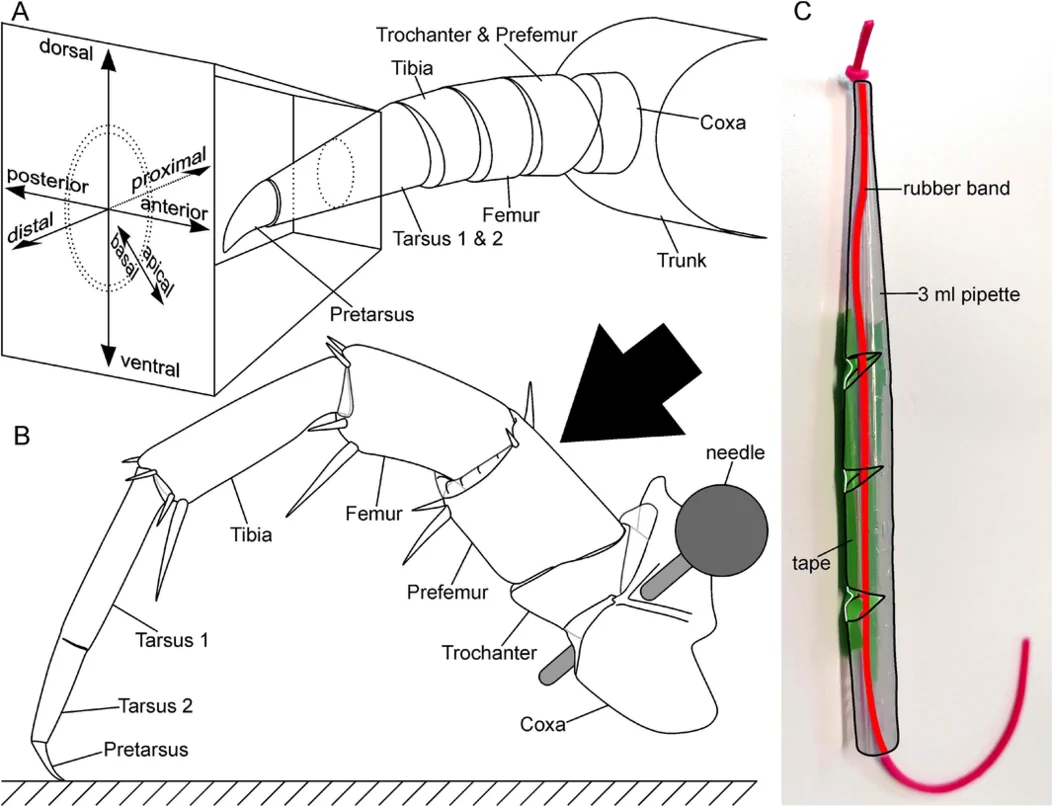
Leg model and clarification of directional information in the leg. (A) Schematic drawing of the right leg of *Lithobius forficatus*, including an enlarged cross‐section and directional information, which will be referred to in the following sections in relation to the legs. (B) Schematic drawing of the contraction imitating experimental setup with the right leg of *L. forficatus* (seen from anterior). A needle fixes the coxa rotatably in a wax dish. Force was applied to the dorsal side of the prefemur with forceps (black arrow). (C) Photograph of the model of a myriapod leg constructed from a plastic pipette.

Directional terms such as anterior, posterior, distal, proximal, dorsal, and ventral refer to the positions of attachment points on the podomeres. Although leg rotation can obscure these orientations, the typically anterior–posteriorly flattened shape of the legs and their alignment to the body allow consistent interpretation (Figure 2A).

All descriptions and names refer to morphemes sensu Richter and Wirkner (2014) and do not imply homology. Muscle coloration is used solely to facilitate visual distinction.

### 2.9 Experiments

#### 2.9.1 Dissection Experiments

The species *L. forficatus* (Chilopoda) and *Spirostreptus* sp. 1 “Tanzania” (Diplopoda) were selected due to their taxonomic position and similar leg size. Specimens were euthanized in a jar containing ethyl acetate‐soaked cloth, inducing muscle relaxation and flaccid death (Adeyemi et al. 2006). Subsequently, specimens were transferred to 1× PBS to prevent dehydration and to maintain osmotic conditions.

Legs were manually bent using forceps, and extension performance was recorded through a Stemi 305 (Zeiss) using a Huawei P30 Lite. Due to variability in applied force and angle, only qualitative observations were possible. Additionally, artificial pressure was applied externally, and the extension response was documented. This procedure was repeated while water was injected into the body cavity at approximately constant pressure.

For further analysis, legs were dissected, and the extension performance of intact joints was observed. The proximal podomere of each joint was fixed with a needle or forceps, and the joint's extension performance was monitored. To eliminate the effects of hemolymph pressure, the anterior and posterior podomeres were then sectioned transversely at mid‐length. Subsequently, the joint membranes were incised ventrally, and extension performance was observed again.

#### 2.9.2 Contraction Simulation Experiments

A mechanical leg model was constructed from a 3 ml plastic pipette (Figure 2C). The closed end was removed, and triangular cutouts were created ventrally to simulate hinge joints. Adhesive tape was applied over the gaps to mimic joint membranes and prevent hyperextension. A rubber band inserted through the pipette simulated muscle contraction when pulled. Extension experiments were conducted with the mechanical leg model, but also with dissected legs of *L. forficatus* and *Spirostreptus* spec. 1 “Tanzania” fixed in 70% Ethanol, whereby the contraction of the extrinsic retractors and intrinsic depressors was simulated by pushing the prefemur downwards (Figure 2B). Due to the fixation, the muscles shortened and the hinge joints flexed. The coxa was fixated using forceps, or it was pierced with a needle near the rotational axis of the coxa–trochanter joint, which was then inserted vertically into a wax dish filled with 1× PBS.

## 3 Results

### 3.1 Walking Leg Morphology

#### 3.1.1 *Scutigera coleoptrata* (Chilopoda)

**Figure 3:**
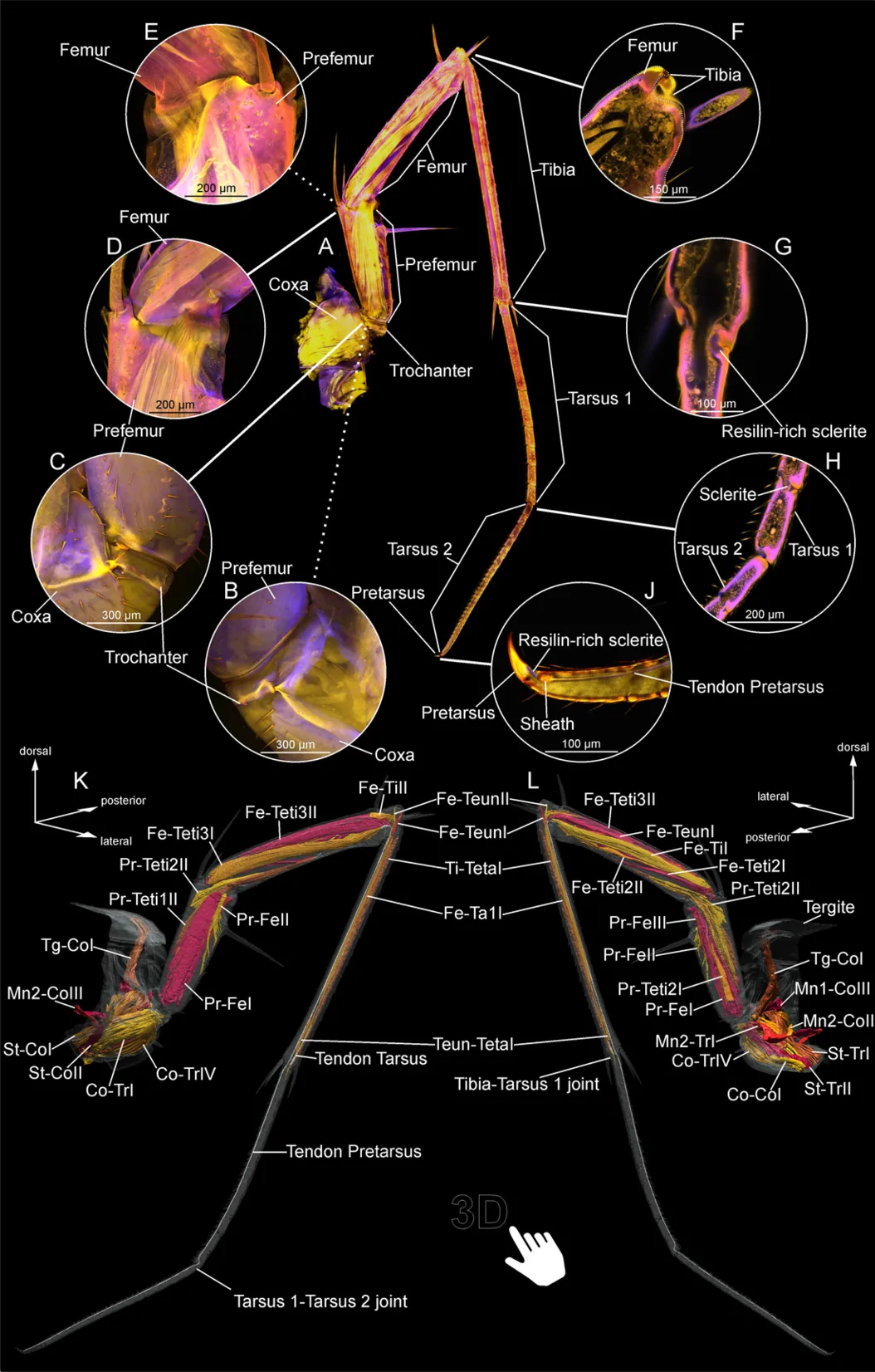
Joint and muscle anatomy of *Scutigera coleoptrata*. (A–E) cLSM maximum intensity projections. (A) Whole left 9th leg, anterior view. (B) Coxa–trochanter joint, posterior view. (C) Coxa–trochanter joint, anterior view. (D) Prefemur–femur joint, anterior view. (E) Prefemur–femur joint, posterior view. (F–J) cLSM longitudinal sections. (F) Femur–tibia joint, anterior view. (G) Tibia–tarsus joint, anterior view. (H) Tarsus 1–tarsus 2 joint, anterior view. (J) Tarsus 2–pretarsus joint, anterior view. (K and L) 3D‐reconstruction of the intrinsic and extrinsic muscles of a left 9th leg. (K) Anterior view. (L) Posterior view. Specimen‐ID (A–H): cLSM 36. Specimen‐ID (J): cLSM 39. Specimen‐ID (K and L): cLSM 36 in combination with µCT Scu015. The PDF version contains interactive 3D content of (K and L). To activate, click on the figure in Adobe Reader.

The legs of *S. coleoptrata* (Linnaeus, 1758) consist of a coxa, trochanter, prefemur, femur, tibia, a multi‐unit tarsus 1 and tarsus 2, and the pretarsus. The coxa is articulated dorsally to the body wall with the epicoxa (see 3D implementation in Figure 3) and is embedded ventrally, as well as anteriorly and posteriorly, in the soft body wall. Distally, the coxa articulates with the trochanter, where an anterior and a posterior ledge of the coxa, running in an anterior–posterior direction, engage with the respective grooves of the trochanter. The trochanter is rigidly and inflexibly connected to the prefemur, which features a ring‐shaped groove encircling the contact point. Distally, the prefemur articulates with the femur. Both the prefemur and the femur have an anterodorsal ridge that runs in an anterior–posterior direction, engaging with corresponding pits in the other podomere, with the ridge of the prefemur lying further dorsally than that of the femur. In contrast, there is no clearly defined joint posterodorsally; a blunt outgrowth of the prefemur loosely grips a cavity in the femur, a joint shape referred to here as a pseudodicondylic hinge joint. The femur engages distodorsally with the tibia, with anterior and posterior ledges of the femur engaging in corresponding pits in the tibia, resulting in a dicondylic hinge joint. Distally, the tibia exhibits an S‐shaped curvature in longitudinal section at the contact point with tarsus 1 (Figure 3G) and articulates with its counterpart, which is also curved in an S‐shape. A notable putatively resilin‐rich region is located ventrally at the contact point, forming a U‐shape at the base of the joint (Figure 3G). The joints between the tarsomeres of tarsus 1 feature a dorsal contact area, with successive tarsomeres connected by anterior and posterior sclerites located in the arthrodial membrane, which were mainly excited by the 488 nm laser (Figure 3H). No such sclerites were found in the tarsus 1–tarsus 2 joint. The joints between the tarsomeres of tarsus 2 resemble those of tarsus 1 proximally but differ distally. There, the sclerites shorten, resulting in two contact points—an anterior and a posterior—while the dorsal contact point is lost. A putatively resilin‐rich sclerite is located ventrally between tarsus 2 (more precisely, the sheath) and the pretarsus. The contact point is situated dorsally, making this joint a hinge joint.

The cuticle at the contact points is primarily excited by light with a wavelength of 633 nm, indicating sclerotization. No particularly strong autofluorescent properties in the excitation range of 405 nm have been found in the arthrodial membranes, which are located basally to the contact points. This indicates that the friction surfaces of the contact points of all joints, except the tarsus 2–pretarsus joint, are located on the outer side of the cuticle.

#### 3.1.2 *L. forficatus* (Chilopoda)

**Figure 4:**
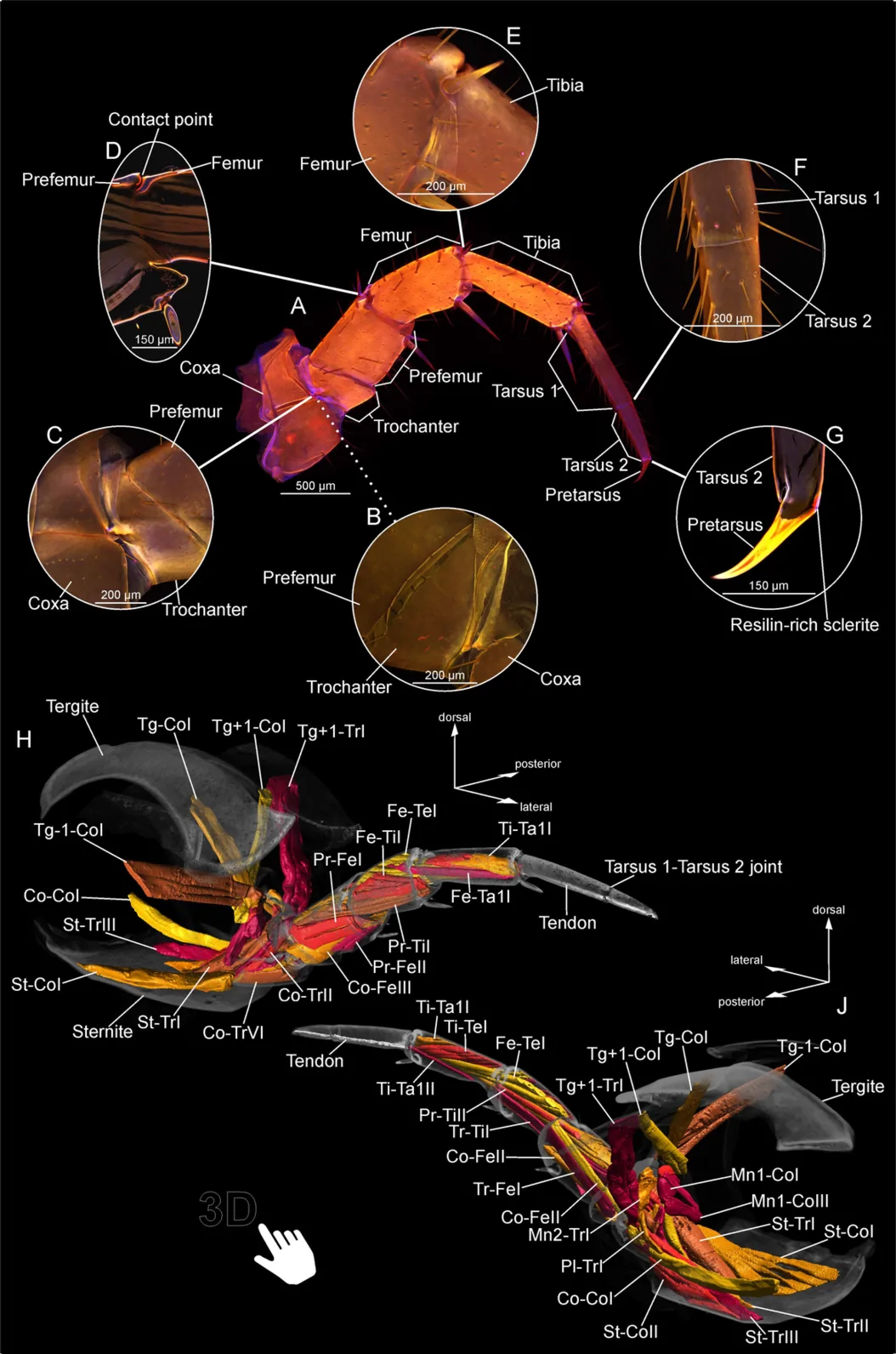
Joint and muscle anatomy of *Lithobius forficatus*. (A–C), (E) ad (F) cLSM maximum intensity projection. (A) Whole left 9th leg, anterior view. (B) Coxa–trochanter joint, posterior view. (C) Coxa–trochanter joint, anterior view. (D) cLSM longitudinal section through the prefemur–femur joint, anterior view. (E) Femur–tibia joint, anterior view. (F) Tarsus 1–tarsus 2 joint, anterior view. (G) cLSM longitudinal section through the tarsus 2–pretarsus joint, anterior view. (H and J) 3D‐reconstruction of the intrinsic and extrinsic muscles of a left 9th leg. (H) Anterolaterodorsal view. (J) Posteromediodorsal view. Specimen‐ID (A–G): cLSM 40. Specimen‐ID (H and J): µCT Lfo001. The PDF version contains interactive 3D content of (H and J). To activate, click on the figure in Adobe Reader.

The legs of *L. forficatus* consist of a coxa, trochanter, prefemur, femur, tibia, tarsus 1, tarsus 2, and pretarsus. A prominent sclerotized longitudinal fold, known as the costa coxalis, is located on the anterior side of the coxa (Figure 4C). Distally, the coxa articulates with the trochanter, forming a dicondylic pivot joint, where the anterior contact point is significantly more complex than the posterior one. The distal margin of the coxa is folded inward anteriorly, as is the proximal edge of the trochanter. Dorsal to this fold lies a putatively resilin‐rich element that is positioned between the edges of the podomeres. The posterior contact point of the joint consists of a pointed outgrowth of the coxa that fits into a small dent on a blunt outgrowth of the trochanter (Figure 4B). No distinct joint between the trochanter and prefemur has been identified, but an arthrodial membrane is present (Figure 4B). True hinge joints are found in the prefemur–femur, femur–tibia, and tibia–tarsus 1 joints, which exhibit strong similarities. The proximal podomeres of those true hinge joints are slightly extended dorsally and engage in a concave dent of the distal podomere. In the longitudinal profile, the proximal contact point shows a slight S‐shape (Figure 4D). The prefemur–tibia joint is slightly displaced anteriorly. A ventral arthrodial membrane exists between tarsus 1 and tarsus 2; however, the cuticle in the dorsal area does not form a differentiated joint but extends continuously across the two tarsomeres. Dorsally, a putatively resilin‐rich sclerite is present in the tarsus 2–pretarsus hinge joint.

In general, sclerotized cuticle is always found at the contact points of the joints. The arthrodial membrane is located basal to the joint in all cases except the tarsus 2–pretarsus joint, which indicates that the friction surfaces of the contact points of all other joints are located on the outer side of the cuticle. These membranes contain a slightly higher proportion of putatively resilin‐like components with autofluorescent properties when excited with a 405 nm laser. This signal is either continuously distributed across the membrane (at the femur–tibia, tibia–tarsus 1, and tarsus 1–tarsus 2 joints) or arranged in longitudinal ridges (posteriorly at the trochanter–prefemur joint and ventrally at the prefemur–femur joint).

#### 3.1.3 *Stigmatogaster subterranea* (Chilopoda)

**Figure 5:**
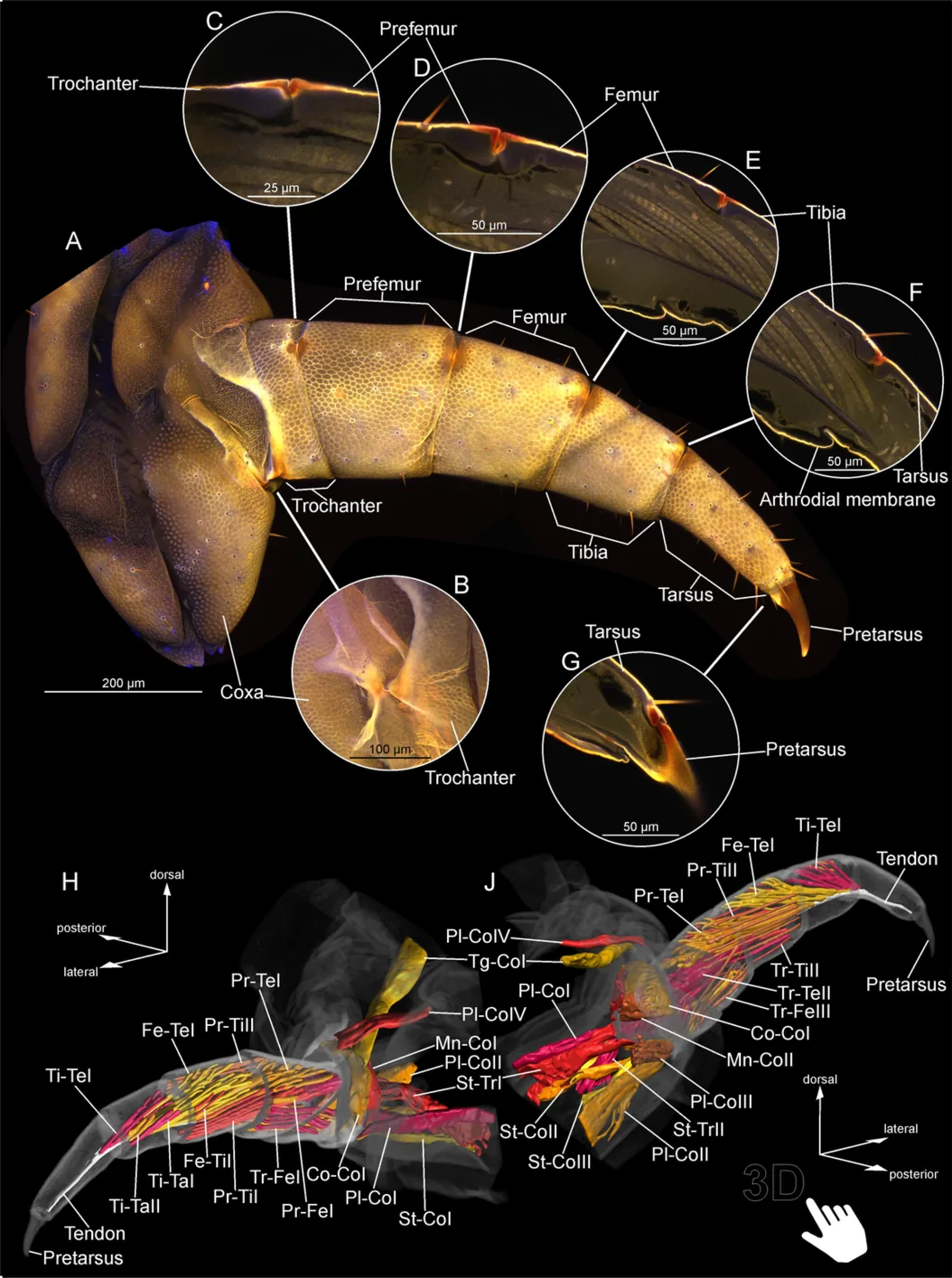
Joint and muscle anatomy of *Stigmatogaster subterranea*. (A and B) cLSM maximum intensity projections. (A) Whole left leg of the mid‐body region, anterior view. (B) Coxa–trochanter joint, anterior view. (C–G) cLSM longitudinal section. (C) Trochanter–prefemur joint, anterior view. (D) Prefemur–femur joint, anterior view. (E) Femur–tibia joint, anterior view. (F) Tibia–tarsus joint, anterior view. (G) Tarsus–pretarsus joint, anterior view. (H and J) 3D‐reconstruction of the intrinsic and extrinsic muscles of the right 2nd leg. (H) Anterolaterodorsal view. (J) Posteromediodorsal view. Specimen‐ID (A): cLSM 45. Specimen‐ID (B–G): cLSM 35. Specimen‐ID (H and J): µCT Ssu01. The PDF version contains interactive 3D content of (H and J). To activate, click on the figure in Adobe Reader.

The legs of *S. subterranea* (Shaw, 1789) consist of a coxa, trochanter, prefemur, femur, tibia, tarsus, and pretarsus (Figure 5A). Anterior at the coxa is the costa coxalis, which is heavily sclerotized and Y‐shaped when viewed from the anterior. One of their three ends projects posteriorly into the coxa and forms the contact point with the trochanter, while another projects dorsally, and the third forms a short apodeme medially. A clearly differentiated joint exists only anteriorly between the coxa and the trochanter. The coxal contact point is slightly outgrown distally, features a small depression apically, and is extended basally into a ridge (see 3D implementation in Figure 5). The contact point of the trochanter extends apically proximally, engages with the depression of the costa coxalis, and runs parallel to it basally. The two ridges of the coxa and the trochanter are connected by a narrow cuticular ligament. The joints between the subsequent podomeres are all characterized as hinge joints and show strong similarities in their structure. Notably, the coxa–trochanter joint is also clearly differentiated as a hinge joint. The dorsal contact points of the joints are strongly sclerotized, and both the proximal and distal podomeres show no differentiation that would allow them to interlock (Figure 5C–F). The exocuticle is thickened in the dorsal area but narrows significantly directly below the contact points of the joint.

In the hinge joint between the tarsus and the pretarsus, there is no resilin‐rich sclerite; however, the contact areas of the joint are heavily sclerotized. In all joints except the tarsus–pretarsus joint, the arthrodial membrane is located basal to the contact points, indicating that the friction surfaces of the contact points of all other joints are located on the outer side of the cuticle. The arthrodial membrane itself is almost undifferentiated and exhibits the same autofluorescent properties as the rest of the podomeres.

#### 3.1.4 *S. subnuda* (Symphyla)

**Figure 6:**
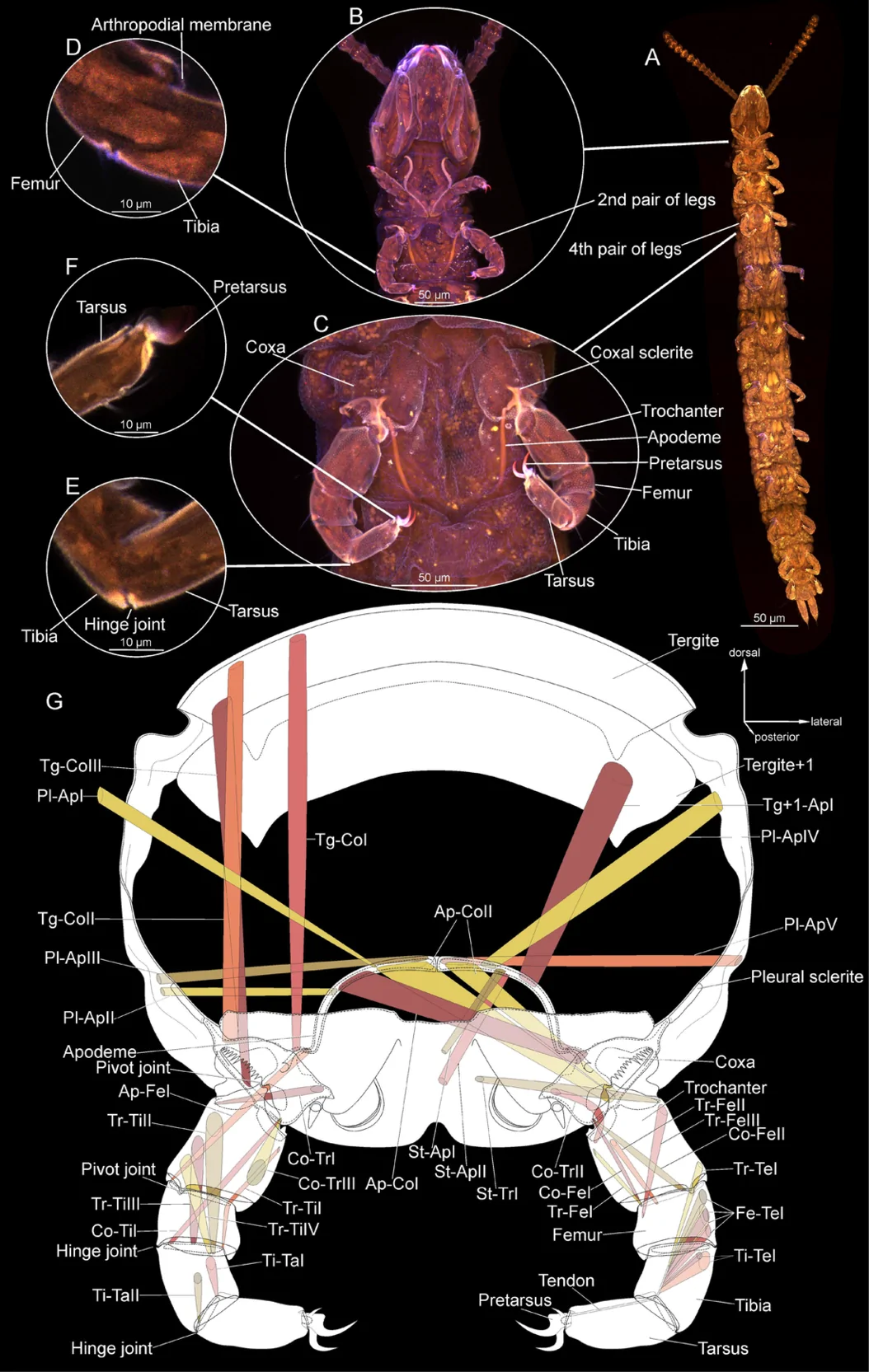
Joint anatomy of *Scolopendrellopsis subnuda* and joint and muscle anatomy of *Scutigerella immaculata.* (A–C) cLSM maximum intensity projection. (A) Whole animal, ventral view. (B) Head and the two first pairs of legs, ventral view. (C) 4th pair of legs, ventral view. (D–F) cLSM longitudinal section. (D) Femur–tibia joint of the left leg of the 2nd pair of legs, anterior view. (E) Tibia–tarsus joint of the left leg of the 4th pair of legs, anterior view. (F) Tarsus–pretarsus joint of the left leg of the 4th pair of legs, anterior view. (G) Drawing of the joint and muscle anatomy, based on a series of semi‐thin sections of *S. immaculata*, anteroventral view. Specimen‐ID (A–F): cLSM 74.

The legs of *S. subnuda* consist of a coxa, trochanter, femur, tibia, tarsus, and pretarsus. Notably, there is a sclerotized sclerite within the coxa. This structure forms the contact points to the trochanter both anteriorly and posteriorly, is closed in a ring shape, connects dorsally to the pleural sclerite, and bears an apodeme that extends significantly posteriorly (Figure 6C,G). The apodeme is an invagination of the anterior coxal wall, making it hollow. The apodemes of one segment converge posteromedially and are connected to each other by connective tissue. Distally, the coxa articulates with the trochanter via a dicondylic pivot joint, where two pointed outgrowths of the coxal sclerite engage in two depressions of the trochanter. These depressions, in turn, lie on two proximal outgrowths of the trochanter. The trochanter–femur joint is also a dicondylic pivot joint, but here, the rotational axis has shifted slightly dorsally. Distally, two knobs extend anterodorsally and posterodorsally from the trochanter, engaging in two depressions of the femur. Both the femur–tibia and tibia–tarsus joints are hinge joints, with their contact points being sclerotized. The contact point of the tibia is slightly curved in an S‐shape in the longitudinal section and fits into a similarly curved contact point of the tarsus. There is no clear dorsal articulation between the tarsus and the pretarsus; instead, the cuticle appears more sclerotized in this area, continuing seamlessly from the tarsus into the pretarsus. A tendon attaches ventrally to the proximal edge of the bipartite pretarsus, which has both an anterior and a posterior claw. This is different from all other investigated species, which possess only one claw.

With the exception of the tarsus–pretarsus joint, the arthrodial membranes also run basal to the contact points, indicating that the friction surfaces of the contact points of all other joints are located on the outer side of the cuticle.

#### 3.1.5 *A. ornatus* (Pauropoda)

**Figure 7:**
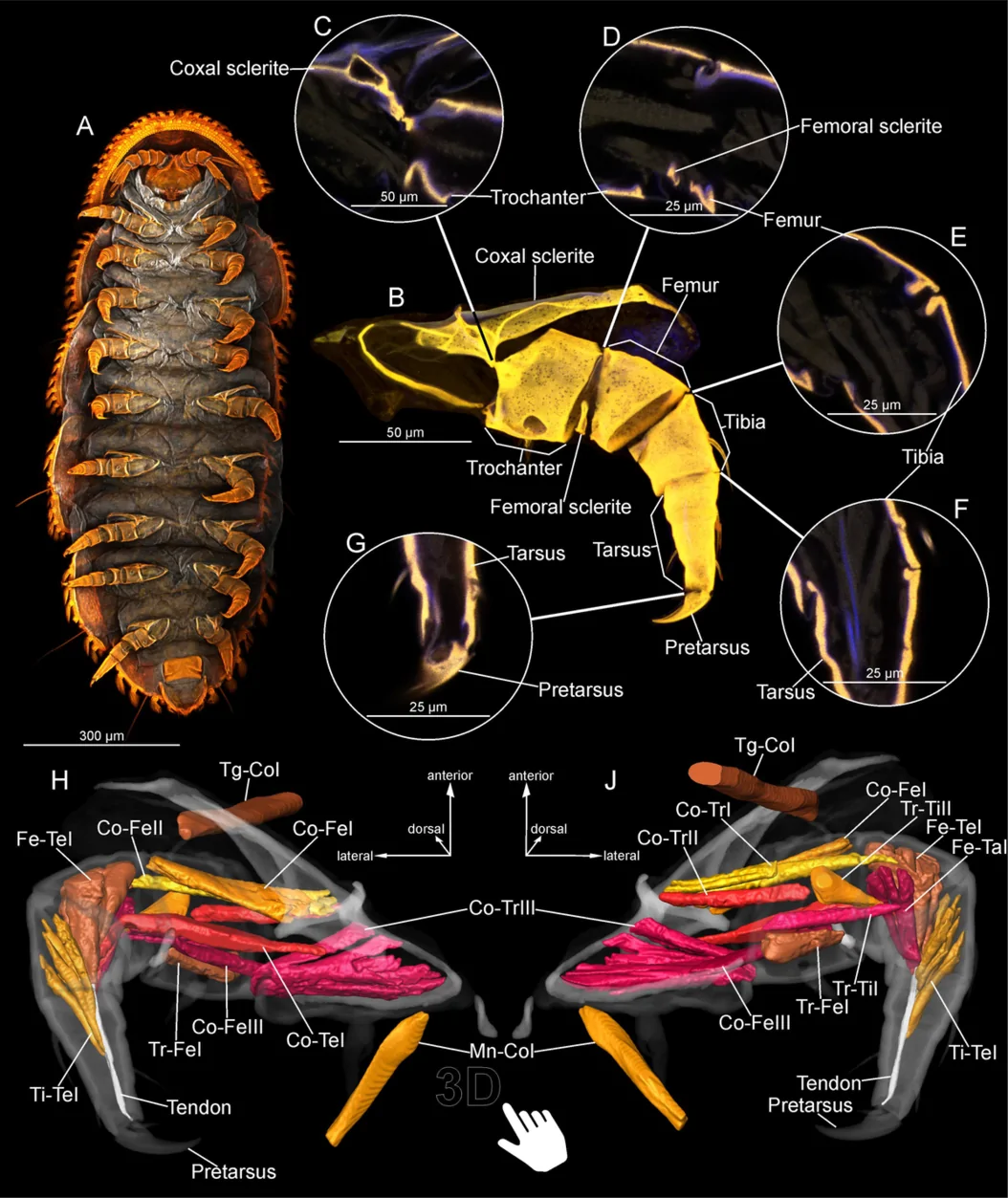
Joint and muscle anatomy of *Acopauropus ornatus*. (A and B) cLSM maximum intensity projection. (A) Whole animal, ventral view, signal excited by laser 405 nm is shown in gray. (B) Whole left leg of the left 3rd pair of legs, anterior view. (C–G) cLSM longitudinal section. (C) Coxa–trochanter joint, anterior pivot point, anterior view. (D) Trochanter–femur joint, anterior view. (E) Femur–tibia joint, anterior view. (F) Tibia–tarsus joint, anterior view. (G) Tarsus–pretarsus joint, anterior view. (H and J) 3D reconstruction of the intrinsic and extrinsic muscles of the right leg of the 4th pair of legs. (H) Anterior view. (J) Posterior view. Specimen‐ID (A): cLSM 72. Specimen‐ID (B–J): cLSM 71. The PDF version contains interactive 3D content of (H and J). To activate, click on the figure in Adobe Reader.

The legs of *A. ornatus* consist of a coxa, trochanter, femur, tibia, tarsus 1, tarsus 2, and pretarsus. Within the coxa, a coxal sclerite is located, which forms an incomplete ring, with a gap extending from the lateral margin to the posterior articulation with the trochanter. The costa coxalis is located anteriorly on the coxal sclerite, where it is the thickest. Apart from the coxal sclerite, the coxa is only weakly sclerotized and is embedded in soft cuticle on the body (Figure 7A,B). Distally, the coxa articulates with the trochanter, with the coxa extending anteriorly and posteriorly to the distal end of the leg and the trochanter extending anteriorly and posteriorly to the proximal end of the leg. The distal outgrowths of the coxa form small depressions at their tips into which the outgrowths of the trochanter fit. The trochanter–femur joint is also a dicondylic pivot joint. The pivot points, slightly displaced dorsally, are formed proximally by outgrowths of the trochanter and distally by shallow depressions of the femur. Directly basal to these articulation points, the arthrodial membrane is clearly sagging and potentially contains resilin, which is indicated by its autofluorescent properties (Figure 7D). Notably, there is a proximoventral part of the femur, referred to here as the femoral sclerite, which is completely surrounded by an arthrodial membrane. Both the femur–tibia and tibia–tarsus 1 joints are distinguished as narrow hinge joints, with the cuticle being heavily thickened at the contact points of both the proximal and distal podomeres (Figure 7E,F). A narrow outgrowth of the proximal podomere engages dorsally in a slight depression of the distal podomere. In *A. ornatus*, the tarsus 1 and tarsus 2 appear to be fused, with only a slight separation of the two tarsomeres noticeable in the dorsal area; however, there is no indication of an arthrodial membrane. The tarsus–pretarsus joint is also a hinge joint, and the cuticle in the contact area does not exhibit a different autofluorescence signal compared to the surrounding sclerotized cuticle, which is consistent across the other joints as well. In all joints, except the tarsus–pretarsus joint, the arthrodial membrane is located basal to the contact points, indicating that the friction surfaces of the contact points of all other joints are situated on the outer side of the cuticle.

#### 3.1.6 *Polyxenus lagurus* (Diplopoda)

**Figure 8:**
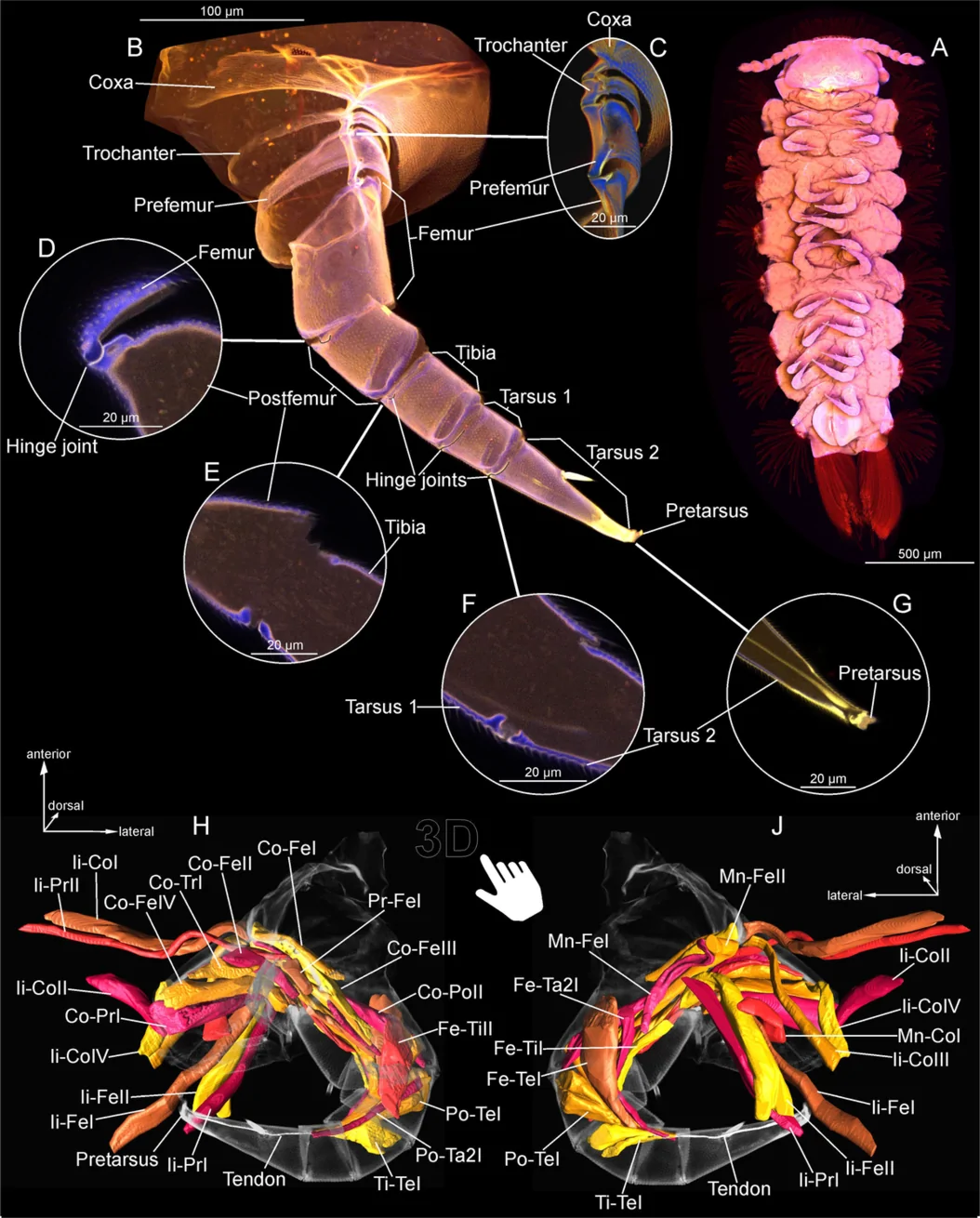
Joint and muscle anatomy of *Polyxenus lagurus*. (A–C) cLSM maximum intensity projection. (A) Whole animal, ventral view. (B) Right leg of the 4th pair of legs, anterior view. (C) Coxa–trochanter, trochanter–prefemur and prefemur–femur joints, anterior parts of the podomeres not shown, anterior view. (D–G) cLSM longitudinal section. (D) Femur–postfemur joint, anterior view. (E) Postfemur–tibia joint, anterior view. (F) Tarsus 1–tarsus 2 joint, anterior view. (G) Tarsus 2–pretarsus joint, anterior view. (H and J) 3D reconstruction of the intrinsic and extrinsic muscles of the left leg of the 3rd pair of legs. (H) Anterior view. (J) Posterior view. Specimen‐ID (A–G): cLSM 26. Specimen‐ID (H and J): cLSM 42. The PDF version contains interactive 3D content of (H), (J), and (B). To activate, click on the figure in Adobe Reader.

The legs of *P. lagurus* consist of a coxa, trochanter, prefemur, femur, postfemur, tibia, tarsus 1, tarsus 2, and pretarsus. The coxa is surrounded by soft, unsclerotized cuticle towards the body and is not fully closed, as a gap extends from the lateral to the posterior side. At the anterior end of the coxa is the costa coxalis, which forms a monocondylic joint with the trochanter. The costa coxalis creates an inwardly protruding ridge, convexly rounded at its distal end, which engages with a concave depression on a similar ridge of the trochanter. The joints between subsequent podomeres follow a similar structure. On the femur, the ridge is formed by an invagination of the anterior cuticle. The arthrodial membrane in these joints is located apically to the contact areas, meaning the contact areas are within the body cavity. Like the coxa, the trochanter is not a fully closed ring but has a posterior opening. The femur–postfemur and postfemur–tibia joints are differentiated as narrow hinge joints. A pronounced dorsal outgrowth of the proximal podomere engages with a well‐defined depression on the distal podomere. The edges of these two structures are almost semicircular (Figure 8D), and the arthrodial membranes are broad on the ventral side. In contrast, the hinge joints between the tibia and tarsus 1, as well as between tarsus 1 and tarsus 2, are wider and less interlocked. In these joints, the cuticle of both the proximal and distal joint regions is thickened around the contact points. Additionally, the ventral arthrodial membrane in these joints is significantly narrower compared to that of the femur–postfemur and postfemur–tibia joints. The pretarsus is also connected to tarsus 2 via a hinge joint, but unlike the other hinge joints, the contact points of this joint are located inside the body cavity.

The tip of tarsus 2 and the pretarsus exhibit a much stronger autofluorescence when excited by a wavelength of 488 nm compared to the rest of the leg. Notably, the cuticle of the leg, except for the aforementioned tip, shows strong autofluorescence under an excitation wavelength of 405 nm.

#### 3.1.7 *Spirostreptus* spec. 1 “Tanzania” sp. and *B. siamensis* (Diplopoda)

**Figure 9:**
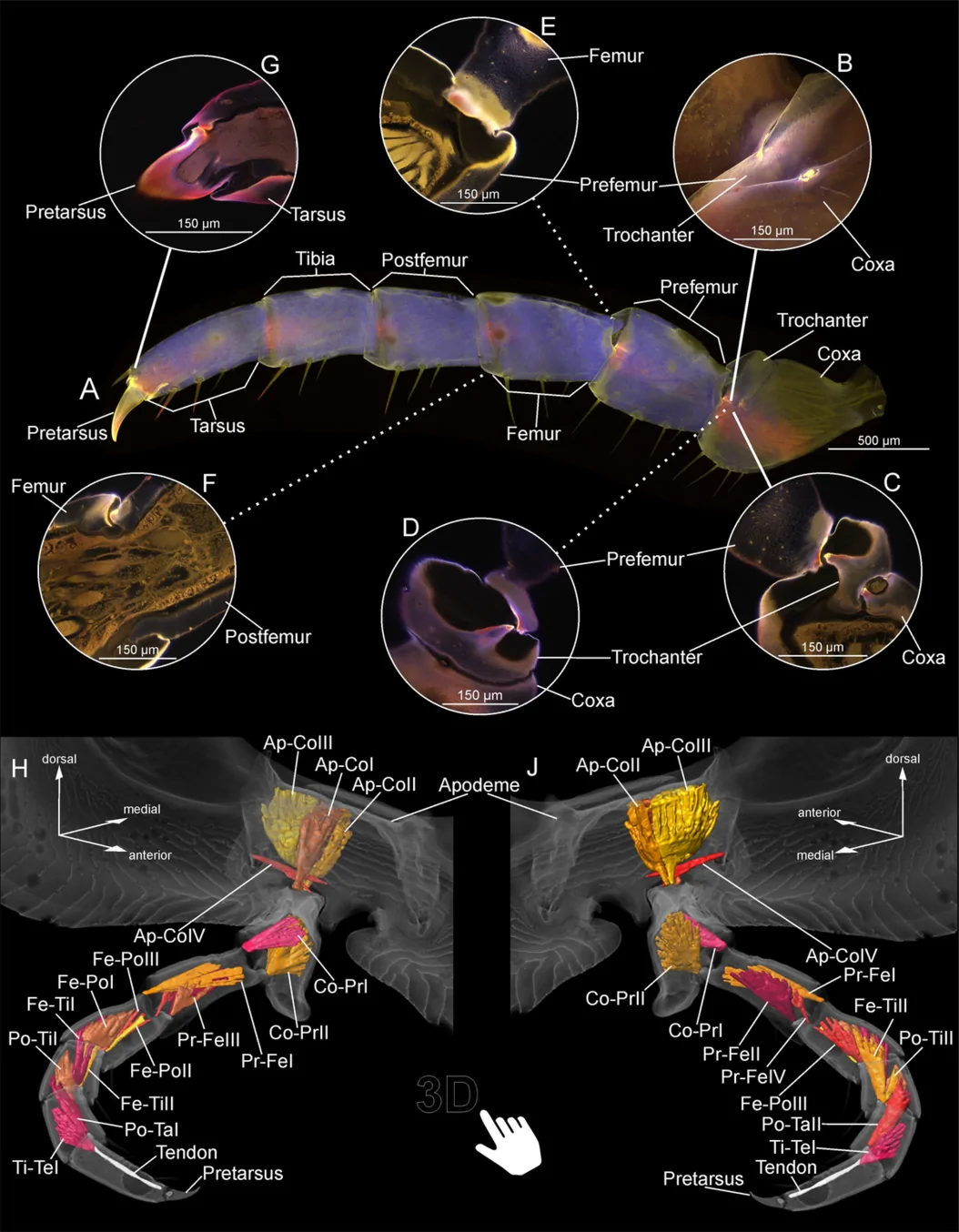
Joint anatomy of *Spirostreptus* spec. 1 “Tanzania” and muscle anatomy of *Benoitolus siamensis*. (A and B) cLSM maximum intensity projections. (A) Whole right leg of the mid‐body region of *Spirostreptus* spec. 1 “Tanzania,” anterior view. (B) Coxa–trochanter and trochanter–prefemur joints, anterior view. (C–G) cLSM longitudinal sections. (C) Coxa–trochanter and trochanter–prefemur joints, anterior view. (D) Coxa–trochanter and trochanter–prefemur joints, posterior view. (E) Prefemur–femur joint, posterior view. (F) Femur–postfemur joint, posterior view. (G) Tarsus–pretarsus joint, posterior view. (H and J) 3D reconstruction of the intrinsic and extrinsic muscles of the right 5th leg of *B. siamensis*. (H) Anterolateral view. (J) Posteromedial view. Specimen‐ID (A–G): cLSM 64. Specimen‐ID (H and J): Bsi01. The PDF version contains interactive 3D content of (H and J). To activate, click on the figure in Adobe Reader.

The legs of *Spirostreptus* spec. 1 “Tanzania” and *B. siamensis* consist of a coxa, trochanter, prefemur, femur, postfemur, tibia, tarsus, and pretarsus. The coxa is significantly narrowed proximally and connects to the fused cuticular body ring via a dicondylic pivot joint. In *B. siamensis*, there is a ventral cut‐out in the cuticle of the body ring to accommodate the coxae of the legs. A ventromedial outgrowth extends from anterior to posterior and is joined laterally by a smaller outgrowth that fits into a depression on the medial coxal wall. Additionally, a larger, more prominent outgrowth extends from lateral to medial, engaging a proximolateral depression on the coxa. A posterolateral flap of the fusion ring also makes contact with the coxa at its posterolateral wall (3D implementation in Figure 9). In *Spirostreptus* spec. 1 “Tanzania,” the coxa articulates distally with the trochanter via a monocondylic joint located anteriorly. No distinct articulation point is discernible posteriorly (Figure 9D). An anterior outgrowth of the coxa, situated relatively far basally, loosely engages in a large recess of the trochanter. The surrounding arthrodial membrane is relatively narrow. The trochanter connects to the prefemur through a dicondylic pivot joint. Two horizontal ridges on the trochanter's anterior and posterior sides engage loosely with depressions on the prefemur, forming a dicondylic pivot joint. The prefemur–femur joint is similar to the trochanter–prefemur joint on the anterior side but lacks a posterior contact point, making it a monocondylic joint. The femur–postfemur, postfemur–tibia, and tibia–tarsus joints are constructed similarly, all functioning as hinge joints with their contact points shifted slightly posteriorly. Both the prefemur and femur are extended proximoventrally, allowing them to interlock with the podomeres proximally adjacent to them. The tarsus–pretarsus joint is also a hinge joint, with the dorsal arthrodial membrane enveloping the apical contact point, which, like all other joints, appears sclerotized. In the hinge joints' contact areas, but not in the monocondylic or dicondylic joints, the entire contact surface is filled with cuticle (Figure 9F). Calcification is present in the exocuticle, indicated by autofluorescence excited with the 405 nm laser, but is absent in the joint membranes and between the contact points.

### 3.2 Extrinsic and Intrinsic Muscles of the Legs

The descriptions of all extrinsic and intrinsic muscles of the investigated species can be found in Appendix S1: Tables 5–10. Illustrations and 3D implementations of the muscles can be found in Figures 3, 4, 5, 6, 7, 8, 9.

### 3.3 Tendon Anatomy

#### 3.3.1 S. coleoptrata

A total of five tendons were identified in *S. coleoptrata*, three of which attach ventrally at the proximal edge of the tibia. Two of these (referred to as tendon tibia 1 and 2) extend proximally into the prefemur. One is very short and terminates in the distal third of the femur (tendon tibia 3). The fourth tendon attaches anteriorly to the proximal edge of tarsus 1, extending proximally almost to the proximal end of the tibia (tendon tarsus). The fifth tendon (tendon pretarsus) attaches to a sheath at the distal end of tarsus 2, which appears to be immovably connected ventrally to tarsus 2 (3D implementation in Figure 3).

#### 3.3.2 *L. forficatus*, *S. subterranea*, *S. immaculata*, *A. ornatus*, *P. lagurus*, *B. siamensis*

Only one tendon is present in the walking legs of *L. forficatus*, *S. subterranea*, *S. immaculata*, *A. ornatus*, *P. lagurus*, and *B. siamensis*. It attaches distally on the ventral side of the proximal margin of the pretarsus. Proximally, it extends differently across the various taxa, and a different number of muscles are attached to it (see Section 3.2).

### 3.4 Experimental Results

#### 3.4.1 Dissection Experiments

##### 3.4.1.1 L. forficatus

After the animals (three individuals) were euthanized, relaxation of the body and extension of the legs could be observed. Even after the legs were bent several times, they extended within an estimated 0.15 s. When pressure was applied to the sternites and the legs were flexed again, they extended at a similar speed. The same behavior was noted when water was injected into the body with a syringe at constant pressure. Remarkably, even after the legs were dissected from the body, they extended at a similar speed to that of the freshly euthanized, intact animals.

Depending on the strength of the pressure applied, squeezing the proximal podomeres with forceps led to either the extension or hyperextension of the legs. It was also observed that artificially hyperextended joints would flex back into the extended position. When the podomeres proximal to a joint were secured with needles, rapid extension of the distal joints was observed. This rapid extension was also noted in isolated joints that were isolated via transversal cuts at the distal and proximal podomeres (Figure 9A).

Additionally, the rotational axis did not appear rigid but could be easily altered by moving the distal podomere anteriorly or posteriorly. However, after the arthrodial membranes were intersected and the podomeres remained connected only at their dorsal contact points, almost no extension occurred.

##### 3.4.1.2 *Spirostreptus* spec. 1 “Tanzania”

The freshly euthanized animals (two individuals) exhibited leg extension similar to that of *L. forficatus*. They also extended rapidly within an estimated average of 0.2 s after being bent several times with forceps. Neither the bending of the animals nor the injection of water, associated with an increase in internal pressure of the body, altered their extension behavior. After the legs were dissected from the body, they continued to extend at a high speed; however, a decrease in extension speed was observed after repeated bending. Notably, when the coxa, which is significantly narrowed proximally, internally and externally, was dissected, an abrupt drop in the rate of extension was recorded, taking on estimated average 6 s to reach the extended position. Through dissections, isolated hinge joints did not extend.

#### 3.4.2 Contraction Imitating Experiments

**Figure 10:**
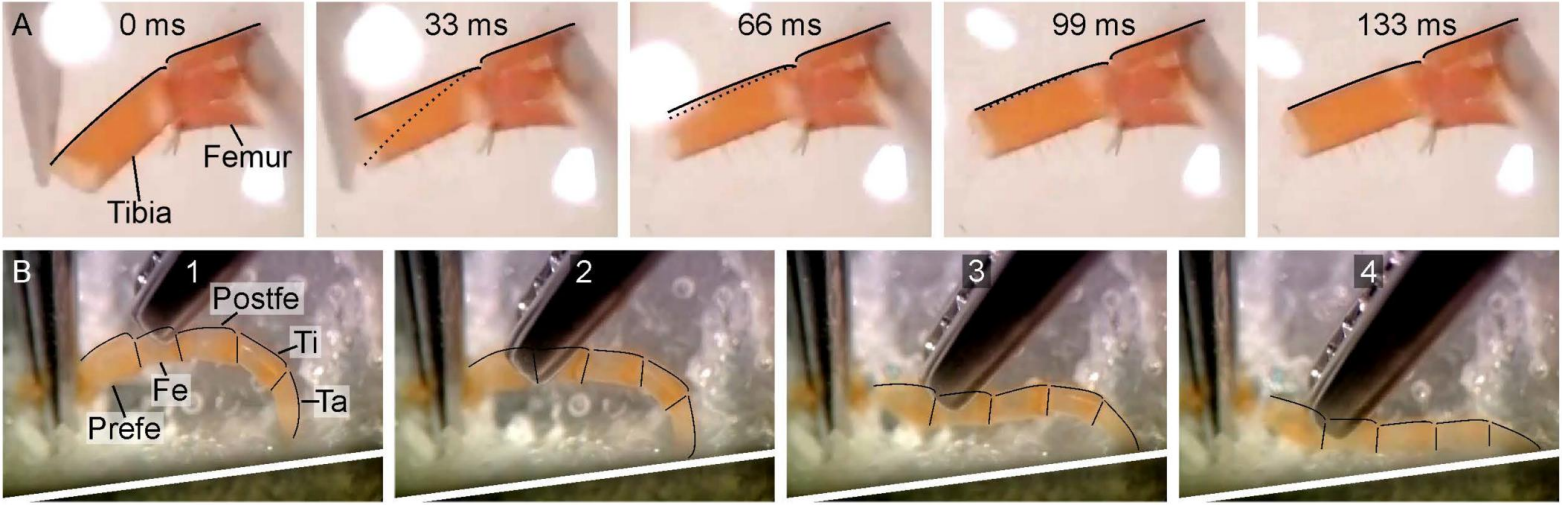
Frames from video documentation of extension experiments. (A) Dissection experiment. The dissected and isolated femur–tibia joint of *Lithobius forficatus* was artificially bent, and its extension behavior was observed. The joint extended multiple times within an estimated average duration of 0.15 s. (B) Contraction‐imitating experiment. The leg of *Spirostreptus* sp. 1 “Tanzania” was fixed at the coxa using forceps, and pressure was artificially applied to the prefemur. Consequently, the tip of the leg was pressed against a flat surface. During this process, the joints opened consecutively from proximal to distal.

The hinge joints of dissected legs of *L. forficatus* and *Spirostreptus* spec. 1 “Tanzania” extends successively from proximal to distal. When pressure is applied to the prefemur from the dorsal side, the prefemur–femur joint is extended. After partial or complete extension, the next distal joint, the femur–postfemur (*Spirostreptus* spec. “Tanzania”) or femur–tibia joint (*L. forficatus*), was extended. This continued until all joints were fully extended (Figure 10B). The experiment with the leg model delivered the same results.

## 4 Discussion

### 4.1 Revision of Walking Leg Morphology Across Myriapoda

#### 4.1.1 Muscles

**Figure 11:**
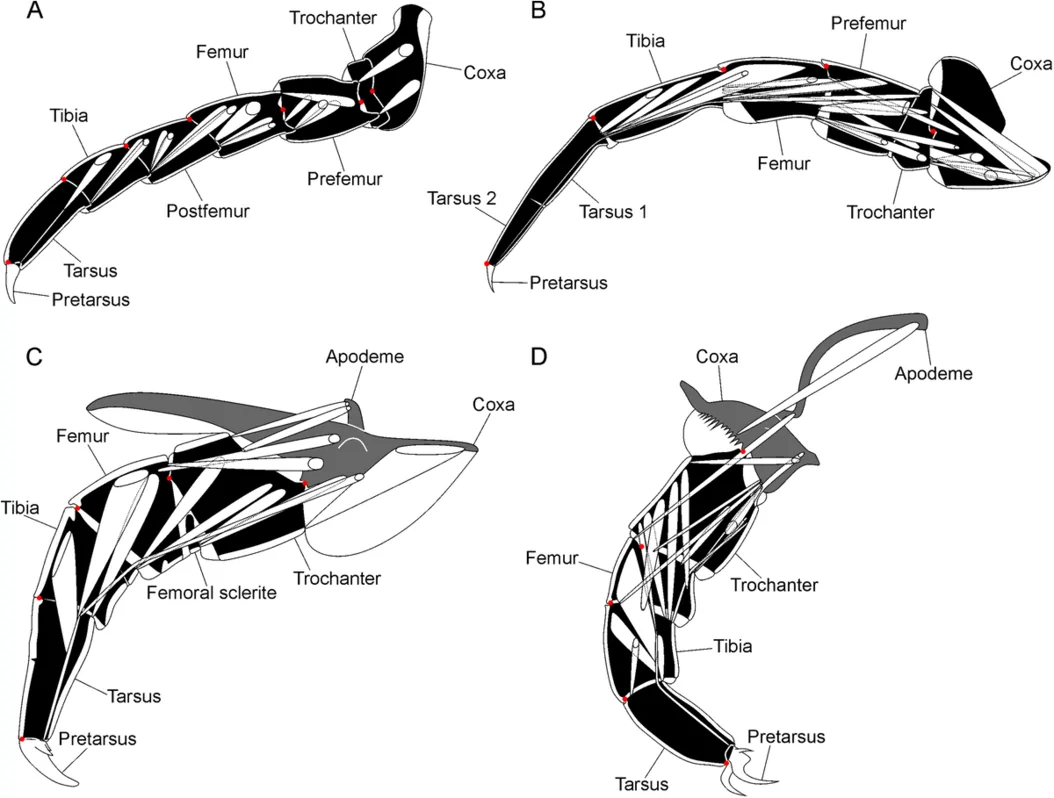
Drawings of the right walking legs of all four major myriapod taxa, viewed from anterior. (A) *Benoitolus siamensis* (Diplopoda). (B) *Lithobius forficatus* (Chilopoda). (C) *Acopauropus ornatus* (Pauropoda). (D) *Scutigerella immaculata* (Symphyla). Podomeres shown in black are cut longitudinally and viewed from the inside; podomeres shown in gray are viewed from the outside. Muscles are illustrated over the cuticle for clarity, but are actually located beneath the gray cuticle. Red dots mark the axes of rotation at the joints.

The legs and leg muscles of Myriapoda have been the focus of numerous studies (e.g., Verhoeff 1934; Tiegs 1940, 1947; Manton 1954, 1957, 1958b, 1961, 1965, 1966; Füller 1963; Rilling 1968; Kenning et al. 2019), yet only a few works have taken a comparative approach to draw a bigger picture of myriapod legs (e.g., Manton 1973). The long‐standing hypothesis that distal joints lack intrinsic extensor muscles (Ewing 1928; Tiegs 1947; Manton 1954, 1957, 1958b, 1961, 1965, 1966; Snodgrass 1958)—and therefore cannot be extended by intrinsic musculature (Manton 1958a, 1965)—was confirmed for all species examined here (Figure 11). Not all muscles described in this study could be matched unambiguously to previously reported ones, largely due to differing descriptive conventions and definitions of muscle individuality. Nevertheless, several previously undescribed intrinsic and extrinsic muscles were identified, including Co‐TrII, Pl‐ApII, Tr‐TiIII, and Ap+1‐CoI in *S. immaculata*. In addition, the leg musculature of species not previously investigated—*A. ornatus*, *S. subterranea*, and *B. siamensis*—was documented. Interactive 3D models were generated for all species except *S. immaculata* (Figures 3K, 4H, 5J, 7H, 8H, 9H).

#### 4.1.2 Cuticle

The structure of the leg cuticle and its joints has been described in detail by Manton for representatives of all four major myriapod taxa (e.g., Manton 1954, 1958b, 1965, 1966). Züger et al. (2024) used cLSM to compare forcipules and walking legs of several chilopods, though without focusing on joint morphology. The data presented here identify potentially resilin‐rich areas and sclerites, providing new insights into joint anatomy across the examined species. For the first time, we describe a potentially resilin‐rich dorsal sclerite in the tibia–tarsus 1 joint of *S. coleoptrata*. We also provide the first descriptions of the leg joints, femoral sclerite, and coxal sclerite in *A. ornatus*, and document a new coxal sclerite in the symphylan *S. subnuda*. In addition, we examined the proximal joints of *P. lagurus* (Figure 8B,C), whose unusual morphology has long been noted but not previously described in detail (Silvestri 1903; Reinecke 1910; Manton 1957). The embedding of contact points within the cuticle of hinge joints in *Spirostreptus* sp. 1 “Tanzania” (Figure 9F) was previously reported for other chilognathan diplopods (Manton 1958b), but our data add new information on the autofluorescent properties of these joints.

#### 4.1.3 Joints Along Walking Legs Exhibit a High Diversity Between Myriapod Taxa

**Figure 12:**
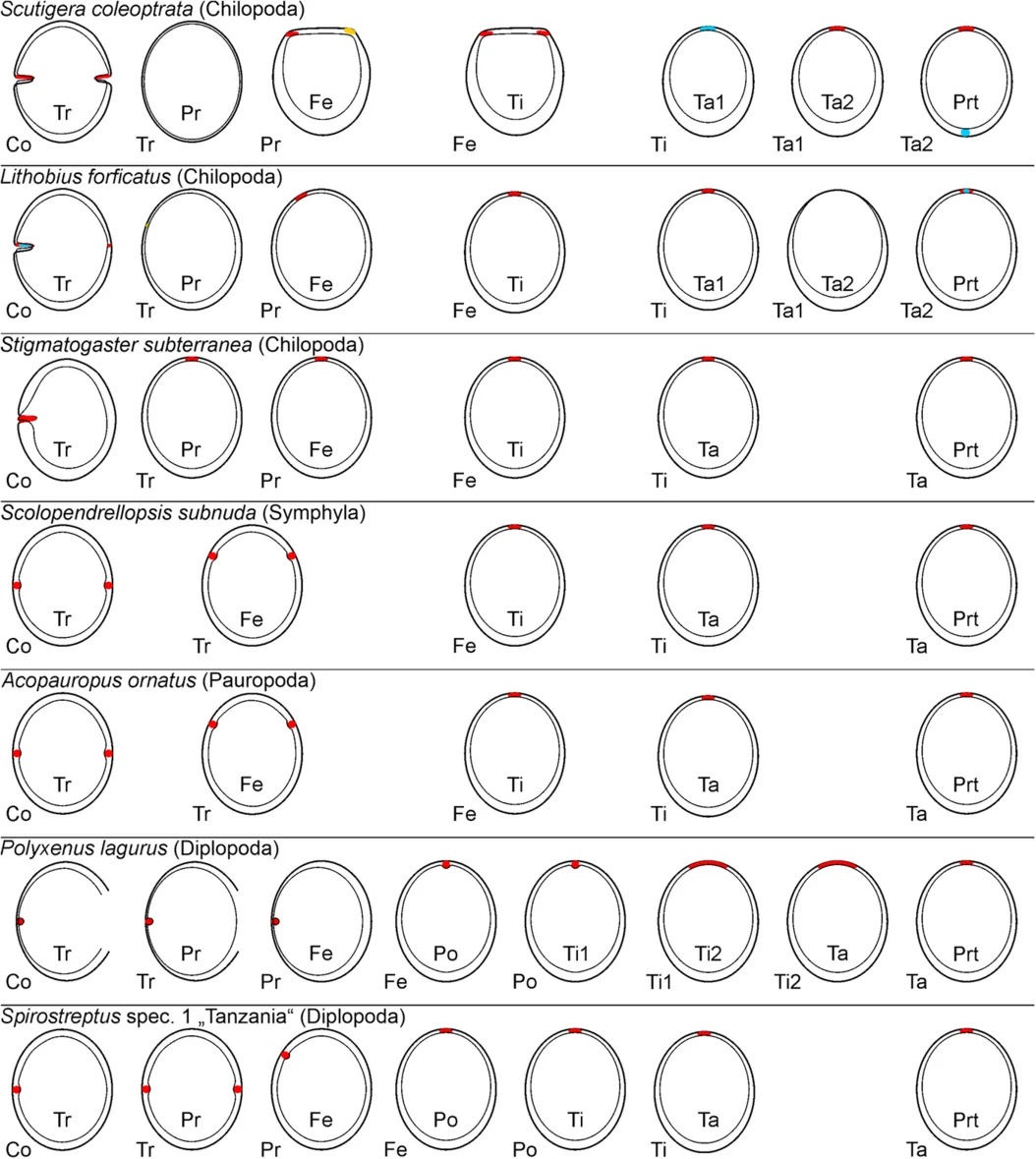
Intra‐ and interspecific comparison of joint anatomies. Schematic representations of cross‐sections through the joints are shown. The outer of the two ellipses represents the proximal podomere, labeled in the lower left corner, while the inner ellipse, labeled inside the ellipse, represents the distal podomere of the joint. Red areas indicate clearly differentiated contact points, while yellow areas indicate only slightly differentiated contact points. Blue dots mark potentially resilin‐rich sclerites. The left side of each joint faces anteriorly, and the upper side faces dorsally.

A comparison of joints along the walking leg across myriapod taxa shows that most species exhibit a largely uniform hinge joint morphology along the leg, including *L. forficatus*, *S. subterranea*, *S. subnuda*, *A. ornatus*, and *Spirostreptus* sp. 1 “Tanzania” (Figure 12). In contrast, *S. coleoptrata* displays pronounced variation along the leg (Figures 3 and 12): the prefemur–femur joint forms a pseudodicondylic hinge (described as dicondylic by Manton 1965), the femur–tibia joint is dicondylic, and the tibia–tarsus 1 joint is a monocondylic hinge joint. Distal secondary tarsomere joints progressively lose their hinge‐like character toward the leg tip.

Marked differences also occur along the leg of *P. lagurus*. The femur–postfemur and postfemur–tibia joints are extremely narrow, leading to tight interlocking of podomeres, whereas the tibia–tarsus 1 and tarsus 1–tarsus 2 joints have broad, loosely connected contact points (Figure 8B,D,F; 3D model; Figure 12). Understanding this diversity will require detailed analyses of joint loading and movement during locomotion.

Across myriapods, joint types vary extensively along the leg. Both proximal and distal joints (e.g., in *S. coleoptrata*) appear to have undergone multiple morphological transformations, affecting not only specific structural details but also the overall articulation type. Due to differences in podomere and joint number, muscle arrangement, and the unresolved phylogeny of Myriapoda (see Section 4.3), no transformation series can currently be reconstructed, yet a graphical overview is provided in Figure 12.

#### 4.1.4 Hypothesis on the Origin of Podomers and Homologization in *P. lagurus*

**Figure 13:**
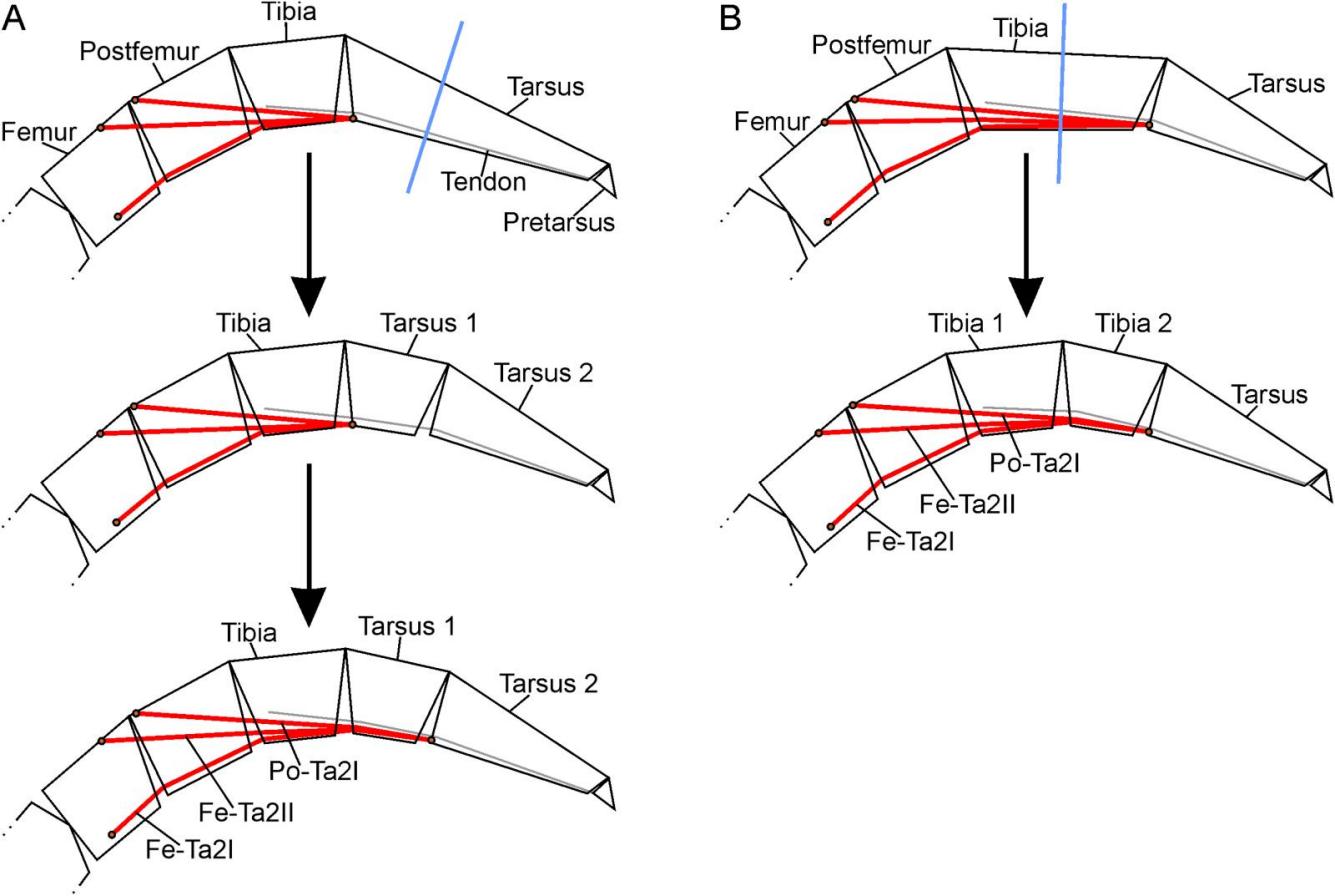
Comparison of two hypotheses on podomere homology in *Polyxenus lagurus*. (A) Hypothesis based on the terminology used in the literature (e.g., Manton 1957). The top images show a potential leg of an ancestor of *P. lagurus*, whose podomere has not yet undergone secondary division. In this hypothesis (A), the tarsus divides, meaning that the distal attachment points of three muscles would shift from the proximal end of tarsus 1 to the proximal end of tarsus 2. (B) New hypothesis. If the tibia, rather than the tarsus, were divided, the distal attachment points of the three muscles would not have had to shift.

The secondary division of the tarsus is found in various taxa within the Myriapoda (e.g., Manton 1954, 1958b, 1965; Boxshall 2004; Kenning et al. 2019). It is striking that no musculature is attached to the now distally located tarsomere but passes through the tarsus 1–tarsus 2 joint in *L. forficatus* and *S. coleoptrata*, as is the case with most secondary annulations (Boxshall 2004). This is different in *P. lagurus*. Here, muscles attach to the proximal edge of tarsus 2, but none to tarsus 1, and they pass through the tibia–tarsus 1 joint. If the tarsus 1–tarsus 2 joint were a secondary division of the tarsus, the Fe‐Ta2I, Fe‐Ta2II, and Po‐Ta2I muscles would have had to shift their distal attachment points from tarsus 1 to tarsus 2. However, if the tibia had been divided into tibia 1 and tibia 2, no muscle insertion site would have shifted (Figure 13). Since the second hypothesis is more parsimonious, we propose that the tarsus did not split secondarily; rather, it is the tibia that was secondarily divided. A similar secondary division of the tibia also occurred, for example, in Amblypygi due to the formation of numerous intratibial joints (Shultz 1999).

### 4.2 Different Mechanisms Contribute Distinctly to Walking Leg Extension in Different Myriapod Taxa

Manton (1958a) proposed that hemolymph pressure contributes to hinge‐joint extension in myriapods, analogous to spiders (e.g., Parry and Brown 1959; Wilson 1970; Anderson and Prestwich 1975; Weihmann et al. 2012; Runge and Wirkner 2019). She rejected joint elasticity as a factor because no elastic sclerites had been identified in myriapod joints, unlike in scorpions (Alexander 1958). Our results, however, indicate that elastic energy storage may occur in the arthrodial membranes of *L. forficatus*, similar to the mechanism described for scorpion chelae (Alexander 1967). Overall, extrinsic and intrinsic hemolymph pressure and joint elasticity seem to contribute to leg extension to different degrees across myriapod taxa.

**Figure 14:**
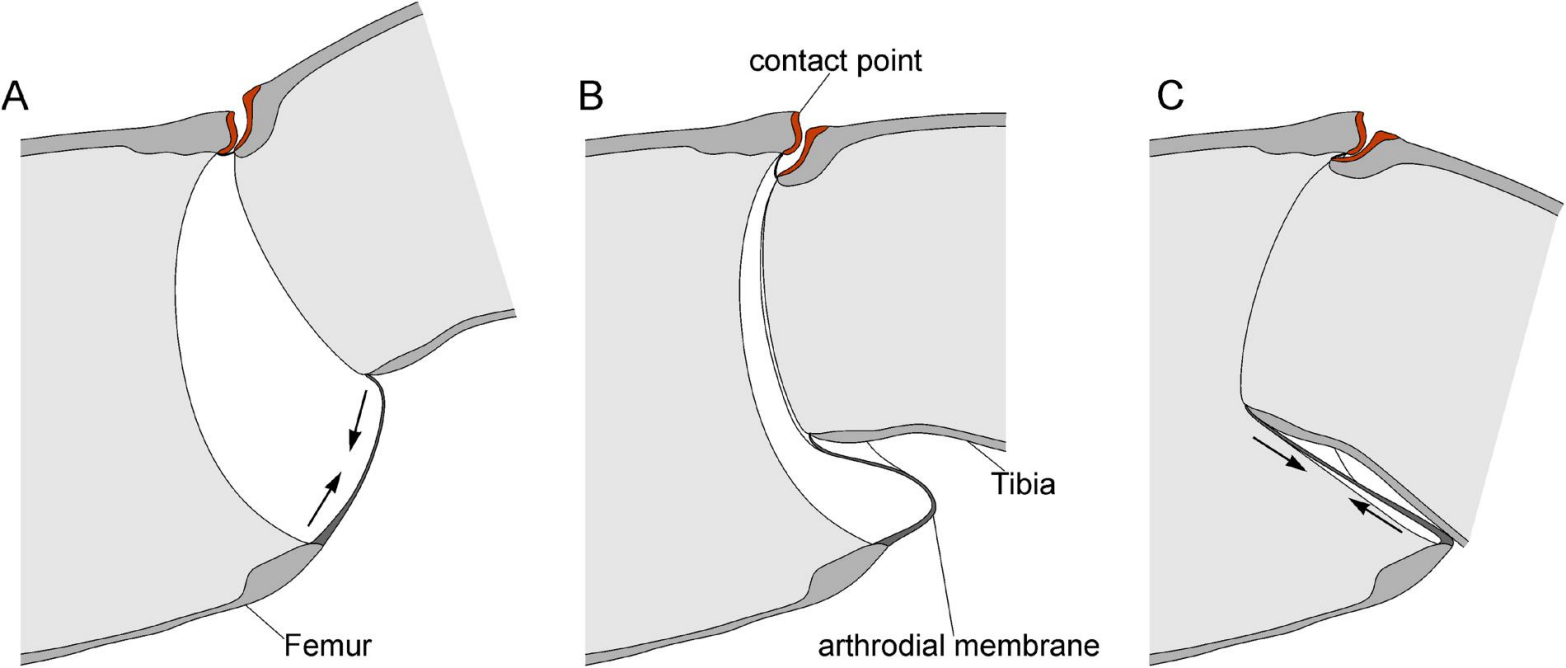
Hypothesis on the hinge joint extension mechanism due to the elasticity of the arthrodial membrane. Longitudinal sections through the femur–tibia joint of *Lithobius forficatus*. (A) Hyperextended position. (B) Resting position. (C) Flexed position. The arthrodial membrane is pulled when the joint goes into hyperextended and flexed positions. This removes its fold. During this transformation, elastic energy might be stored in the arthrodial membrane, which, when relaxed, causes the fold to form again. During this formation, the ventrodistal edge of the femur and the ventroproximal edge of the tibia are pulled towards each other (black arrows).

In *L. forficatus*, legs extend passively after flaccid death and even after being dissected proximal to the coxa, revealing that trunk‐generated hemolymph pressure is presumably not the sole driver. Local pressure applied to proximal podomeres caused distal hinge joints to extend, indicating that intrinsic, locally increased hemolymph pressure likely contributes to extension during flexion. Increasing overall hemolymph pressure by pressing on the freshly killed animal had little effect, although such manipulation cannot replicate natural pressure conditions. A combined effect of intrinsic and extrinsic pressure remains possible. Even when legs were further dissected to eliminate artificial pressure increases (Section 2.9.1), they extended at similar speeds to intact legs (Figure 10A). This suggests that a substantial portion of the extension force derives from joint elasticity. Cutting the arthrodial membrane caused a sharp reduction in extension speed, supporting the conclusion that tension in the ventral membrane generated during flexion contributes to elastic recoil (Figure 14). CLSM data corroborate this: no potentially resilin‐rich sclerites occur dorsally in the hinge joints, but an indication for slightly higher resilin density was observed in the arthrodial membranes. Regions of cuticle regions, presumably low in resilin, were consistently flexible and resilient, indicating that elastic tension arises not only from resilin but also from other cuticular components.

Freshly killed specimens of *Spirostreptus* sp. 1 “Tanzania” showed fully extended legs, and repeated bending resulted in rapid re‐extension, indicating at least partial passive extension. Trunk‐generated hemolymph pressure did not noticeably affect extension speed while having a noticeable effect on gonopod erection (unpublished observation). After complete leg removal, extension persisted at similar speeds, but removing the coxa caused a drastic drop. Combined with the strongly narrowed proximal coxa (Figure 9A), this suggests that local hemolymph pressure generated during flexion is the primary driver of extension in this species. Because intrinsic pressure is eliminated when the coxa is removed, and extension becomes slow, joint elasticity appears to play only a minor role. CLSM scans revealed no increased content of putative resilin in the arthrodial membranes or dorsal hinge‐joint regions, consistent with the hypothesized low contribution of elasticity.

**Figure 15:**
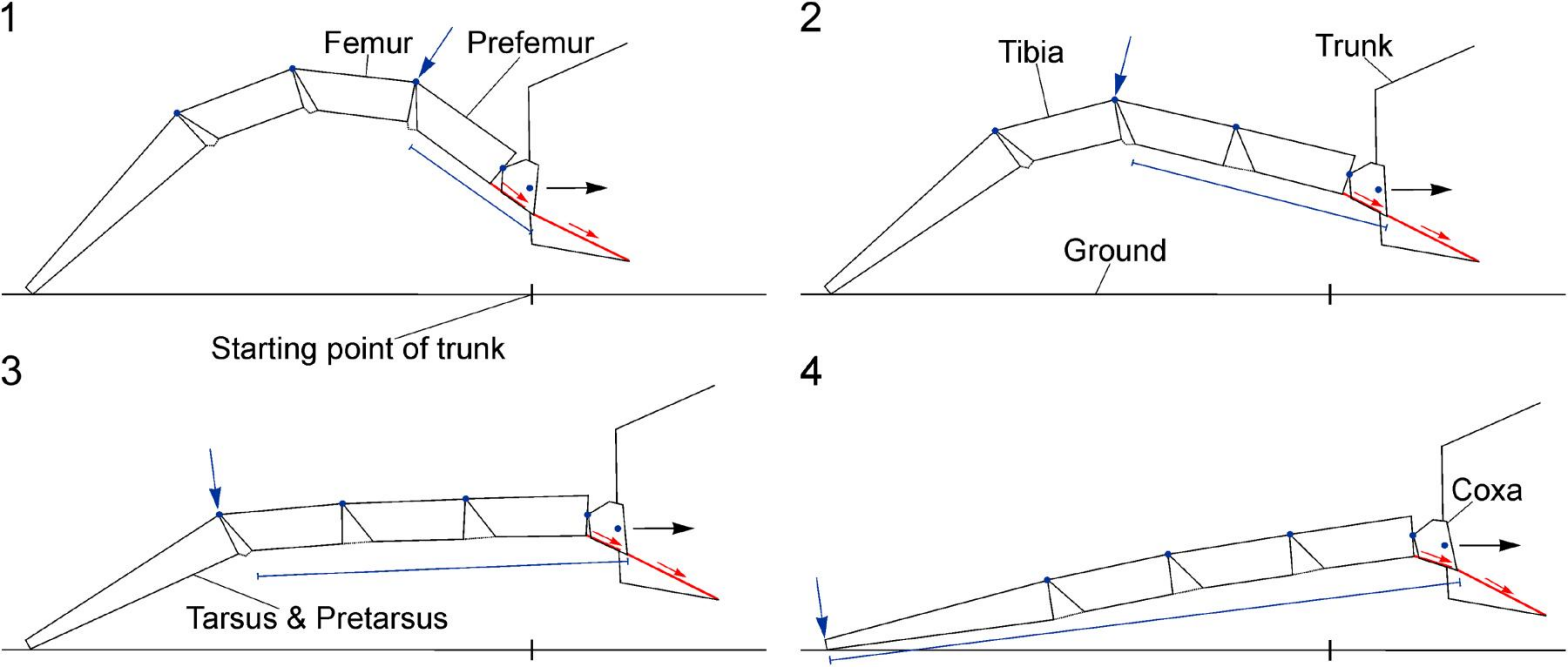
Hypothesis on the leg extension mechanism by extrinsic retractors and intrinsic depressors in *Lithobius forficatus*. The contraction of the intrinsic depressors, along with simultaneous contraction of the extrinsic retractors (red arrows), pulls the coxa back‐ and downwards, and the distal end of the prefemur downward. The force is transmitted vertically to the prefemur–femur joint (1) (blue arrow). This joint extends until the arthrodial membrane is tightened, or until the intrinsic flexors contract, preventing further extension. As a result, the podomere distal to the now‐extended joint extends the lever arm transmitting the force generated by muscle contraction (2). The force can then be applied to the next distal joint (3), continuing until all hinge joints are fully extended (4). As the leg extends, propulsion might be generated (black arrow). The blue bar indicates which podomeres function as a lever arm and transmit force. This drawing deliberately omits the influence of the intrinsic flexors to present the mechanism in isolation. Presumably, coordinated contraction of the intrinsic flexors can produce a uniform extension. Moreover, in living animals, this mechanism does not result in complete joint extension (see text).

Another extension mechanism involves pressing the leg tip against the substrate during the second half of the propulsive backstroke (Manton 1958a, 1965). The extrinsic retractor and proximal intrinsic depressors generate the required force. Contraction‐imitating experiments on dissected legs of *L. forficatus*, *Spirostreptus* sp. 1 “Tanzania,” and a mechanical leg model (Figure 2C) support this mechanism. Ventral displacement of the prefemur increases the ventral prefemur–femur angle until the hinge‐joint membrane limits extension (Figures 10 and 15). Prefemur and femur act as a lever arm transmitting force to the femur–tibia/postfemur joint, sequentially extending all hinge joints. As the lever arm lengthens, force transmission becomes less efficient distally, and effective leg length increases, potentially generating propulsion. In vivo, this mechanism alone does not fully extend the joints (Manton 1958a).

We propose that flexor‐muscle contraction at specific force levels stiffens the hinge joint at certain extension angles, enabling more uniform force transfer through the kinematic chain of podomeres. A comparable mechanism has been described for large spiders (Weihmann et al. 2012).

A further hypothesis involves elastic energy storage in dorsal sclerites, as in Solifugae (Sensenig and Shultz 2004). A U‐shaped, potentially resilin‐rich sclerite was found in the tibia–tarsus joint of *S. coleoptrata*, suggesting a similar mechanism that requires experimental confirmation. No comparable structures were found in other joints.

The relative importance of extension mechanisms differs markedly between the two studied taxa. In *Spirostreptus* sp. 1 “Tanzania,” intrinsic hemolymph pressure combined with pressing the leg tip against the substrate appears to dominate. In *L. forficatus*, leg extension results from a more complex interplay of hemolymph pressure, elastic energy storage in the arthrodial membrane, and pressing the leg tip against the substrate.

### 4.3 Methodological Limitations

The experiments conducted here provide only circumstantial evidence and require further, including quantitative, studies for confirmation. These should include analyses of joint resistance at various degrees of flexion and under different hemolymph pressures, similar to the approach used by Sensenig and Shultz (2004) in solifuges. The presence of resilin should also be verified using pH‑dependent autofluorescence (Neff et al. 2000) or antibody labeling (Burrows et al. 2011). Furthermore, it cannot be ruled out that during the experiments, substantially greater forces were expected on the legs than would occur in a living animal. To assess this, force measurements on living specimens (as, i.e., Reinhardt et al. 2009 did with ants) and high‑speed recordings of legs in motion would be required (as, i.e., Boehm et al. 2021 did with spiders). However, conducting such experiments lies beyond the scope of this study and must be addressed in future studies.

### 4.4 Biological Significance of Joint and Muscle Flexibility of Walking Legs of Myriapoda

Our experiments show that the hinge joints of *L. forficatus* are not fixed but can partially shift, unlike those of *Spirostreptus* sp. 1 “Tanzania.” All joints—especially the tibia–tarsus 1 joint—allow substantial movement outside the anterior—posterior rotational axis. Manipulating the distal podomere can markedly alter the alignment of the rotational axis. Manton (1965) interpreted this as unwanted instability requiring muscular stabilization (e.g., Pr‐TiII, Fe‐TiI, Ti‐Ta1I, Ti‐Ta1II). We propose instead that this flexibility may be adaptive. *L. forficatus* inhabits leaf litter, rotting wood, and the space beneath bark (Scheu 2017) and can reach high running speeds when hunting (Manton 1952). In such confined habitats, yielding joints likely reduce the risk of injury or entrapment when encountering obstacles.

In contrast, the joints of *S. coleoptrata* do not permit displacement of rotational axes. This increases the efficiency of force transmission along the kinematic chain, as no energy is lost through plastic deformation. Because *S. coleoptrata* typically occupies open spaces (Scheu 2017), the risk of legs catching on obstacles is low. Many Spirostreptidae also inhabit ground litter but are slow detritivores (Mwabvu 2017). Their reduced speed, short legs, and stabilizing calcified cuticle further minimize injury risk. In these taxa, less flexible joints enhance power transmission, which is advantageous for the bulldozer‐like locomotion characteristic of many diplopods (Manton 1954).

This stability is partly achieved by positioning the hinge joint contact point deep within the cuticle and by the proximal podomere enclosing the proximoventral end of the distal podomere, both of which likely contribute to increased joint stability.

### 4.5 Phylogenetic Implications

Regardless of the current phylogeny of the Myriapoda, several observations can be made about the ground pattern of this taxon. In all species studied, including representatives from all four major myriapod taxa, hinge joints were found in the distal joints that cannot be extended by intrinsic musculature. This holds true for all Myriapoda species studied to date, with the exception of *Platyrhacus* sp. (Manton 1958b), where the intrinsic muscular extension mechanism of some hinge joints has evolved most likely secondarily. It is therefore highly probable that hinge joints, which are not driven by intrinsic extensor muscles, were already present in the ground pattern of Myriapoda. However, the transformation of the proximal joints and what can be inferred from this about the ground pattern strongly depend on the underlying phylogeny. The traditional Progoneata–Dignatha hypothesis (Edgecombe 2004; Giribet et al. 2005; Gai et al. 2008) has been called into question in recent years by various phylogenetic studies (Miyazawa et al. 2014; Rehm et al. 2014; Wang et al. 2021; Benavides et al. 2023). The new phylogenies contradict each other, which is why a further discussion of the ground pattern is not included.

## 5 Conclusion

In this study, new insights into the cuticle and muscle anatomy of Myriapoda were gained and visualized using 3D models. A new hypothesis regarding the secondary division of the podomeres in *P. lagurus* was formulated. Furthermore, various mechanisms contributing to the extension of the hinge joints were hypothesized, including intrinsic hemolymph pressure, the pressing of the tips of the legs against the substrate by extrinsic retractor and intrinsic depressor muscles, as well as the elasticity of the joint membranes in *L. forficatus*. The hypothesis of hinge joints being present in the ground pattern of Myriapoda is further supported by the current findings.

## Author Contributions

**Birk Rillich:** investigation, resources, validation, visualization, writing – original draft preparation. **Benjamin Naumann:** conceptualization, investigation, resources, writing – review and editing. **Tom Weihmann:** conceptualization, investigation, supervision, writing – review and editing. **Christian S. Wirkner:** conceptualization, investigation, project administration, methodology, supervision, validation, writing – review and editing.

## Conflicts of Interest

The authors declare no conflicts of interest.

## Declaration of Generative AI and AI‐Assisted Technologies in the Writing Process

During the preparation of this work, the authors used ChatGPT to improve the English with regard to grammar, style, and spelling. After using this service, the authors reviewed and edited the content as needed and take full responsibility for the content of the publication.

## Supporting information


Supporting File

## Data Availability

The datasets used and/or analyzed during the current study are available from the corresponding author on reasonable request.
