# Supplementary material for: Extending Without Extensors: Morphofunctional Insights and Qualitative Experiments on Myriapod Legs
Source: J Morphol. 2026 Sep 23;287(9):e70171. doi: 10.1002/jmor.70171 (PMC13598734; doi:10.1002/jmor.70171)
Supplement: Supplementary file 1 [file JMOR-287-e70171-s001.docx]

7 Appendix

**App. Table 1: List of abbreviations.**

| AA | Ascorbic acid |
| --- | --- |
| Ap | Apodeme |
| BABB | Benzoic Acid Benzyl Benzoate |
| cLSM | Confocal laser scanning microscopy |
| Co | Coxa |
| CPD | Critical point drying |
| d | Day |
| DESY | Deutsches Elektronen-Synchrotron |
| Eth | Ethanol |
| Fe | Femur |
| Gly | Glycerine |
| Ii | Intersegmental ventral insertion area |
| Meth | Methanol |
| min | Minutes |
| Mn | Muscle node |
| PBS | Phosphate-buffered saline |
| bPFA | Buffered Paraformaline |
| Pl | Plica |
| Po | Postfemur |
| Pr | Prefemur |
| Prt | pretarsus |
| PTA | Phosphor tungstic acid |
| Ta | Tarsus |
| Ta1 | Tarsus 1 |
| Ta2 | Tarsus 2 |
| Te | Tendon |
| Teta | Tendon tarsus |
| Teti | Tendon tibia |
| Teprt | Tendon pretarsus |
| Ti | Tibia |
| Tg | Tergite |
| Tr | Trochanter |
| St | Sternite |

**App. Table 2: List of species and samples analyzed with the cLSM with the corresponding washing steps of the clearing methods.** Each washing step not provided with a time specification lasted 30 min and was carried out on a shaker at room temperature. AA - Ascorbic acid, Eth - Ethanol, Gly - Glycerine, Meth - Methanol.

**App. Table 3: List of species and samples analyzed with the µCT with the corresponding washing steps of the clearing methods.** Each washing step not provided with a time specification lasted 30 min and was carried out on a shaker at room temperature. CPD - Critical point drying, DESY - Deutsches Elektronen-Synchrotron, Eth - Ethanol.

| Species | *Scutigera coleoptrata* | *Lithobius forficatus* | *Stigmatogaster subterranea* | *Benoitolus siamensis* |
| --- | --- | --- | --- | --- |
| Specimen-ID | Scu015 | Lfo001 | Ssu01 | Bsi001 |
| Fixative | Dubosq basil | Dubosq basil | 4% PFA | 70% Ethanol |
| Contrasting method | Iodine | PTA | Iodine | propagation-based phase contrast |
| Device | Zeiss XRadia Versa 410 | Zeiss XRadia Versa 410 | Zeiss XRadia Versa 410 | DESY |
| Magnification | 4x | 4x | 10x | 10x |
| Voltage (kV) | 50 | 70 | 80 | 33.3 |
| Power (W) | 8 | 8 | 8 |  |
| Washing steps | 70% Eth | 70% Eth | 4% PFA | 70% Eth |
|  | 80% Eth | 80% Eth | Aqua dest. | 80% Eth |
|  | 90% Eth | 90% Eth | 10% Eth | 90% Eth |
|  | 96% Eth + 1% Iodine | 3% PTA in 90% Eth (7 d) | 20% Eth | 96% Eth |
|  | 99.8% Eth + 1% Iodine (2 d) | 3x 99.8% Eth | 30% Eth | 99.8% Eth |
|  | 99.8% Eth (15 min) | CPD | 40%Eth |  |
|  | CPD |  | 50% Eth |  |
|  |  |  | 60% Eth |  |
|  |  |  | 70% Eth |  |
|  |  |  | 80% Eth |  |
|  |  |  | 96% Eth + 1% Iodine |  |
|  |  |  | 99.8% Eth + 1% Iodine (2 d) |  |
|  |  |  | 99.8% Eth (15 min) |  |
|  |  |  | CPD |  |

**App. Table 4: In this study described extrinsic and intrinsic leg muscles in *Scutigera coleoptrata.***

| *Scutigera coleoptrata* | | |
| --- | --- | --- |
| Name of muscle | Insertion point 1 | Insertion point 2 |
| Mn1-CoI | Muscle node located dorsal at the base of the coxa | anterodorsal at the proximal margin of the coxa |
| Mn1-CoII | Muscle node located dorsal at the base of the coxa | anterodorsal at the face of the coxa |
| Mn1-CoIII | Muscle node located dorsal at the base of the coxa | dorsal at the proximal margin of the coxa |
| Mn1-CoIV | Muscle node located dorsal at the base of the coxa | posteroventral at the proximal margin of the coxa |
| Mn1-CoV | Muscle node located dorsal at the base of the coxa | posterodorsal at the proximal margin of the coxa |
| Mn2-CoI | intersegmental muscle node located ventromedian at the anterior end of the segment | anterior at the proximal margin of the coxa |
| Mn2-CoII | intersegmental muscle node located ventromedian at the posterior end of the segment | anterior at the proximal margin of the coxa |
| Mn2-CoIII | intersegmental muscle node located ventrolateral at the anterior end of the segment | anterior at the proximal margin of the coxa |
| Mn2-TrI | intersegmental muscle node located ventromedian at the posterior end of the segment | posteroventral at the proximal end of the trochanter |
| Mn2-TrII | intersegmental muscle node located ventrolateral at the posterior end of the segment | at the trochanter apodeme at the anteroproximal end of the trochanter |
| Mn3-CoI | muscle node located posteroventral at the base of the coxa | posteroventral at the proximal margin of the coxa |
| Mn3-TrI | muscle node located posteroventral at the base of the coxa | posterovenral at the proximal margin of the trochanter |
| St-CoI | anteromedial at the sternite | dorsal at the face of the coxa |
| St-CoII | anterolateral at the sternite | ventromedial at the proximal margin of the coxa |
| St-CoIII | medial at the sternite | ventromedial at the proximal margin of the coxa |
| St-TrI | anteromedial at the sternite | dorsal at the proximal margin of the trochanter |
| St-TrII | posteromedial at the sternite | wide from ventral to posteroventral at the proximal margin of the trochanter |
| Tg-CoI | Slightly lateral at the tergite | posterodorsal at the proximal margin of the coxa |
| Co-CoI | posteroventral at the proximal margin of the contralateral coxa | posteroventral at the proximal margin of the coxa |
| Co-TrI | wide from anterior to anteroventral at the proximal margin to anterior to anteroventral at the face of the coxa | wide from anterior to ventral at the proximal margin of the trochanter |
| Co-TrII | anterior at the proximal margin of the coxa | anterodorsal at the proximal margin of the trochanter |
| Co-TrIII | anteroproximal at the face of the coxa | posteroventral at the face of the trochanter |
| Co-TrIV | wide from proximoventral to posteroproximoventral at the face of the coxa | wide ventral at the proximal margin of the trochanter |
| Co-TrV | anterior at the face of the coxa | anterodorsal at the face of the trochanter |
| Co-TrVI | anterodistal at the face of the coxa | posteroventral at the face of the trochanter |
| Co-TrVII | anterodistal at the face of the coxa, close to the anterior articulation point of the coxa-trochanter joint | wide from dorsal to posterodorsal at the face of the trochanter |
| Pr-FeI | wide from anteroproximodorsal to proximoventral at the face of the prefemur | wide from anterodorsal to ventral at the proximal margin of the femur |
| Pr-FeII | wide from proximoventral to distoventral at the face of the prefemur | anteroventral at the proximal margin of the femur |
| Pr-FeIII | ventral at the face of the prefemur | ventral at the proximal margin of the femur |
| Pr-Teti1I | posteroproximal at the face of the prefemur | tendon tibia 1 |
| Pr-Teti1II | dorsal at the face of the prefemur | tendon tibia 1 |
| Pr-Teti2I | posteroproximal at the face of the prefemur | tendon tibia 2 |
| Pr-Teti2II | wide from dorsal to distodorsal at the face of the prefemur | tendon tibia 2 |
| Fe-TiI | proximodorsal at the face of the femur | ventral at the proximal margin of the tibia |
| Fe-TiII | distodorsal at the face of the femur | anterior at the proximal margin of the tibia |
| Fe-Teti1I | dorsal at the proximal margin of the femur | tendon tibia 1 |
| Fe-Teti1II | proximodorsal at the face of the femur | tendon tibia 1 |
| Fe-Teti2I | proximodorsal at the face of the femur | tendon tibia 2 |
| Fe-Teti2II | proximodorsal at the face of the femur | tendon tibia 2 |
| Fe-Teti3I | dorsal at the proximal margin of the femur | tendon tibia 3 |
| Fe-Teti3II | proximodorsal at the face of the femur | tendon tibia 3 |
| Fe-Ta1I | distodorsal at the face of the femur | proximodorsal at the face of the tarsus 1 |
| Fe-TeprtI | dorsal at the face of the femur | tendon pretarsus |
| Fe-TeprtII | distodorsal at the face of the femur | tendon pretarsus |
| Ti-Teta1I | wide from proximodorsal to distodorsal at the face of the tibia | tendon tarsus |
| Ti-TeprtI | proximodorsal at the face of the tibia | tendon pretarsus |
| Teprt-Teta | distal in the tibia at the tendon pretarsus | more distal in the tibia at the tendon tarsus |

**App. Table 5: In this study described extrinsic and intrinsic leg muscles in *Lithobius forficatus.***

| *Lithobius forficatus* | | |
| --- | --- | --- |
| Name of muscle | Insertion point 1 | Insertion point 2 |
| Mn1-CoI | Muscle node located centrally at the base of the coxa | anteroventral at the proximal margin of the coxa |
| Mn1-CoII | Muscle node located centrally at the base of the coxa | posterodorsal at the proximal margin of the coxa |
| Mn1-CoIII | Muscle node located centrally at the base of the coxa | anteroventral at the proximal margin of the coxa |
| Mn1-CoIV | Muscle node located centrally at the base of the coxa | ventral at the proximal margin of the coxa |
| Mn1-CoV | Muscle node located centrally at the base of the coxa | posteroventral at the proximal margin of the coxa |
| Mn1-CoVI | Muscle node located centrally at the base of the coxa | posteroventral at the proximal face of the coxa |
| Mn1-TrI | Muscle node located centrally at the base of the coxa | anterior at the proximal margin of the trochanter |
| Mn2-CoI | Muscle node located dorsal at the base of the coxa | anterodorsal at the proximal margin of the coxa |
| Mn2-CoII | Muscle node located dorsal at the base of the coxa | anteroventral at the proximal margin of the coxa |
| Mn2-TrI | Muscle node located dorsal at the base of the coxa | posterior at the proximal margin of the trochanter |
| Pl-TrI | Ventrolateral at plica to the next posterior segment | posterior at the proximal margin of the trochanter |
| St-CoI | anterolateral on the opposite side of the sternite | anteroventral at the proximal margin of the coxa |
| St-CoII | posterolateral on the opposite side of the sternite | posteroventral at the proximal margin of the coxa |
| St-TrI | slightly posterior to the center of the sternite | dorsal at the proximal margin of the trochanter |
| St-TrII | slightly posterior to the center of the sternite | posteroventral at the proximal margin of the trochanter |
| St-TrIII | posterolateral on the opposite side of the sternite | posteroventral at the proximal margin of the trochanter |
| St-TrIV | slightly posterior to the center of the sternite | posterior at the proximal margin of the trochanter |
| Tg-CoI | slightly anterolateral to the center of the tergite | dorsal at the proximal margin of the coxa |
| Tg+1-CoI | anterolateral on the tergite located one segment further posterior | anterodorsal at the proximal margin of the coxa |
| Tg+1-TrI | anterolateral on the tergite located one segment further posterior | posterior at the proximal margin of the trochanter |
| Tg-1-CoI | anterolateral on the tergite located one segment further anterior | dorsal tip of the coxa |
| Co-CoI | posteroventral at the proximal margin of the contralateral coxa | posteroventral at the proximal margin of the ipsilateral coxa |
| Co-TrI | wide from ventral to ventrolateral at the posterior margin of the coxa | wide from anterodorsal to posterodorsal at the proximal face of the trochanter |
| Co-TrII | wide from anterior to ventral at the posterior margin of the coxa | posterodorsal at the proximal face of the trochanter |
| Co-TrIII | ventral at the proximal margin of the coxa | posteroventral at the proximal margin of the trochanter |
| Co-TrIV | anteroventral at the proximal margin of the coxa | posteroventral at the proximal margin of the trochanter |
| Co-TrV | anterior at the anterior face of the coxa | posteroventral at the proximal margin of the trochanter |
| Co-TrVI | wide from anterior to ventral at the posterior face of the coxa | wide from anteroventral to ventral at the proximal margin of the trochanter |
| Co-FeI | anterior at the anterior face of the coxa | ventral at the proximal margin of the femur |
| Co-FeII | posteroproximal at the face of the coxa | dorsolateral at the proximal margin of the femur |
| Co-FeIII | wide from anteroproximal to ventral at the face of the coxa | ventral at the proximal margin of the femur |
| Tr-FeI | posteroventral at the face of the trochanter | posterior at the proximal margin of the femur |
| Tr-TiI | posterior at the face of the trochanter | wide from ventral to posterior at the proximal margin of the tibia |
| Tr-TeI | dorsal at the face of the trochanter | tendon |
| Pr-FeI | wide from anterodorsal to anterior at the proximal margin of the prefemur | anteroventral at the proximal margin of the femur |
| Pr-FeII | antoventroproximal at the face of the prefemur | ventral at the proximal margin of the femur |
| Pr-TiI | dorsal at the face of the prefemur | anteroventral at the proximal margin of the tibia |
| Pr-TiII | posterodorsodistal at the face of the prefemur | posterior at the proximal margin of the tibia |
| Pr-TeI | dorsal at the face of the prefemur | tendon |
| Fe-TiI | anterodorsal at the face of the femur | anterior at the proximal margin of the tibia |
| Fe-Ta1I | anterodistodorsal at the face of the femur | anteroventral at the proximal margin of the tarsus 1 |
| Fe-TeI | Wide from proximodorsal to distodorsal at the face of the femur | tendon |
| Ti-Ta1I | anterodorsal at the face of the tibia | anterior at the proximal margin of the tarsus 1 |
| Ti-Ta1II | proximoventral at the face of the tibia | posterior at the proximal margin of the tarsus 1 |
| Ti-TeI | dorsal at the face of the tibia | tendon |

**App. Table 6: In this study described extrinsic and intrinsic leg muscles in *Stigmatogaster subterranea.***

| *Stigmatogaster subterranea* | | |
| --- | --- | --- |
| Name of muscle | Insertion point 1 | Insertion point 2 |
| Mn-CoI | Muscle node located posterodorsal at the base of the coxa | dorsal at the costa coxalis (anteroventral at the proximal end of the coxa) |
| Mn-CoII | Muscle node located posterodorsal at the base of the coxa | posterodorsal at the face of the coxa |
| Pl-CoI | medial at the anterior intersegmental plica | transition between procoxa and costa coxalis (anteroventral at the proximal end of the coxa) |
| Pl-CoII | medioventral at the posterior intersegmental plica | ventral at the face of the coxa |
| Pl-CoIII | lateroventral at the posterior intersegmental plica | ventral at the face of the coxa |
| Pl-CoIV | dorsolateral at the anterior intersegmental plica | dorsal at the proximal margin of the coxa |
| St-CoI | anteromedial at the sternite | transition between procoxa and costa coxalis (anteroventral at the proximal end of the coxa) |
| St-CoII | anteromedial at the sternite | ventral at the face of the coxa |
| St-CoIII | medial at the sternite | ventral at the face of the coxa |
| St-TrI | anteromedial at the sternite and medial at the ventral intersegmental plica | wide from anterodorsal to posterodorsal at the proximal face of the trochanter |
| St-TrII | slightly lateral at the sternite | anterodorsal at the proximal margin of the trochanter |
| Tg-CoI | medial at the tergite | anterodorsal at the proximal margin of the coxa |
| Co-CoI | wide from posterior to dorsal at the face of the coxa | dorsal at the costa coxalis (anteroventral at the proximal end of the coxa) |
| Co-TrI | medioposterior at the costa coxalis | anterior at the proximal margin of the trochanter |
| Co-TrII | medioposterior at the costa coxalis | posteroventral at the proximal margin of the trochanter |
| Co-FeI | posterior end of costa coxalis | ventral at the proximal margin of the femur |
| Tr-PrI | posteroproximal at the face of the trochanter | posterior at the proximal margin of the prefemur |
| Tr-FeI | anterior at face of the trochanter | anterior at the proximal margin of the femur |
| Tr-FeII | posteroventral at the face of the trochanter | ventral at the proximal margin of the femur |
| Tr-FeIII | posteroventral at the face of the trochanter | wide from posterior to ventral at the proximal margin of the femur |
| Tr-TiI | anterior face of the trochanter | ventral at the proximal margin of the tibia |
| Tr-TiII | posterior face of the trochanter | ventral at the proximal margin of the tibia |
| Tr-TeI | anterior at face of the trochanter | tendon |
| Tr-TeII | posterodorsal at the proximal margin of the trochanter | tendon |
| Pr-FeI | anterior at face of the prefemur | anterior at the proximal margin of the femur |
| Pr-TiI | wide from anterior to anterodistal at the face of the prefemur | wide from anterior to ventral at the proximal margin of the tibia |
| Pr-TiII | wide from posterior to posterodistal at the face of the prefemur | wide from posterior to posteroventral at the proximal margin of the tibia |
| Pr-TeI | wide from proximodorsal to distodorsal of the prefemur | tendon |
| Fe-TiI | anterodorsal at the face of the femur | anterodorsal at the proximal margin of the tibia |
| Fe-TaI | anterodorsal at the face of the femur | ventral at the proximal margin of the tarsus |
| Fe-TeI | wide from proximodorsal to dorsodistal of the femur | tendon |
| Ti-TaI | anteroproximodorsal at the surfae of the tibia | anteroventral at the proximal margin of the tarsus |
| Ti-TaII | anterodorsal at the face of the tibia | anterodorsal at the proximal margin of the tarsus |
| Ti-TeI | dorsal at the face of the tibia | tendon |

**App. Table 7: In this study described extrinsic and intrinsic leg muscles in *Scutigerella immaculata.***

| *Scutigerella immaculata* | | |
| --- | --- | --- |
| Name of muscle | Insertion point 1 | Insertion point 2 |
| Pl-ApI | on the pleura slightly anterior to the insertion point on the apodeme | in the middle between the base and the connection point of the apodeme |
| Pl-ApII | on the pleura slightly anterior to the insertion point on the apodeme | at the apodeme close to the connection point to the contralateral apodeme |
| Pl-ApIII | on the pleura dorsal to the insertion point on the apodeme | posterior at the apodeme between insertion points of Pl-ApII and Pl-ApIV |
| Pl-ApIV | on the pleura at the level of the insertion point on the apodeme | at the apodeme close to the connection point to the contralateral apodeme |
| Pl-ApV | anterior at the pleura, anterodorsal in relation to the attachment point on the apodeme | medial at the apodeme |
| St-ApI | medial at the sternite | posteroventral at the apodeme |
| St-ApII | medial at the sternite slightly posterior to the insertion point on the apodeme | posteroventral at the apodeme |
| St-TrI | slightly lateral at the sternite | proximodorsal at the face of the trochanter |
| Tg-CoI | at the tergite directly above the coxa | lateral at the proximal end of the apodeme |
| Tg-CoII | at the tergite directly above the coxa | anterodorsal/-lateral at the proximal margin of the coxa |
| Tg-CoIII | posterolateral at the tergite | posterior at the proximal margin of the coxa |
| Tg+1-Ap | lateral at the tergite of the posterior following segment | in the middle between the base and the connection point of the apodeme |
| Ap-Ap+1I | wide at the apodeme from its base to the middle between its base and the connection point | medial at the apodeme of the posterior segment |
| Ap-CoI | in the middle between the base and the connection point of the contralateral apodeme | anterior at the proximal margin of tha coxa |
| Ap-CoII | wide at the tip of the ipsi- and contralateral apodeme | posterior at the proximal margin of the coxa |
| Ap-FeI | anterior at the proximal end of the apodeme | dorsal at the proximal margin of the femur |
| Ap+1-CoI | in the middle between the base and the connection point of the apodeme of the anterior segment | anterior at the proximal margin of the coxa |
| Co-TrI | medial/ventral at the proximal margin of the coxa | proximodorsal at the face of the trochanter |
| Co-TrII | medial/ventral at the proximal margin of the coxa | ventral at the proximal margin of the trochanter |
| Co-TrIII | ventral at the distal margin of the coxa | wide anteroventral at the face of the trochanter |
| Co-FeI | ventral at the distal margin of the coxa | ventral at the proximal margin of the femur |
| Co-FeII | ventral at the distal margin of the coxa | anterior, slightly ventral of the anterior contact point, at the proximal margin of the femur |
| Co-TiI | ventral at the distal margin of the coxa | anterodorsal at the proximal margin of the tibia |
| Tr-FeI | proximoventral at the face of the trochanter | ventral at the proximal margin of the femur |
| Tr-FeII | posteroproximoventral at the face of the trochanter | posteroventral at the proximal margin of the femur |
| Tr-FeIII | proximodorsal at the face of the trochanter | proximoventral at the face of the femur |
| Tr-TiI | proximoventral at the face of the trochanter | ventral at the proximal margin of the tibia |
| Tr-TiII | posterodorsal at the face of the trochanter | posteroventral at the proximal margin of the tibia |
| Tr-TiIII | posterodorsal at the face of the trochanter | anterior at the proximal margin of the tibia |
| Tr-TiIV | posterodistodorsal at the face of the trochanter | posteroventral at the proximal margin of the tibia |
| Tr-TeI | proximodorsal at the face of the trochanter | tendon |
| Fe-TeI | wide dorsal at the face of the femur | tendon |
| Ti-TeI | posteroproximodorsal at the face of the tibia | tendon |
| Ti-TeII | anteroproximal at the face of the tibia | tendon |
| Ti-TaI | posteroproximoventral at the face of the tibia | ventral at the proximal margin of the tarsus |
| Ti-TaII | anterior at the face of the tibia | dorsoanterior at the proximal margin of the tarsus |

**App. Table 8: In this study described extrinsic and intrinsic leg muscles in *Acopauropus ornatus.***

| *Acopauropus ornatus* | | |
| --- | --- | --- |
| Name of muscle | Insertion point 1 | Insertion point 2 |
| Mn-CoI | Muscle node located posterolateral of the base of the coxa | posteromedioventral at the proximal margin of the coxa |
| Tg-CoI | anterior approximately midway between the center and the lateral border of the tergite | dorsal at the face of the coxa |
| Co-TrI | coxal apodeme | dorsal at the proximal margin of the trochanter |
| Co-TrII | coxal apodeme | anterodorsal at the proximal margin of the trochanter |
| Co-TrIII | wide from ventroanterior to ventral at the coxal sclerite | ventral at the proximal margin of the trochanter |
| Co-FeI | on a ledge located at the front of the coxal sclerite, running in the longitudinal direction of the leg | dorsal at the proximal margin of the femur |
| Co-FeII | on a ledge located at the front of the coxal sclerite, running in the longitudinal direction of the leg | proximodorsal at the face of the femur |
| Co-FeIII | posterolateral at the coxal sclerite | ventral at the proximal margin of the femur |
| Co-TeI | anterior at the coxal sclerite | tendon |
| Tr-FeI | posteroproximal at the face of the trochanter | at the femur sclerite |
| Tr-TiI | posteroproximal at the face of the trochanter | ventral at the proximal margin of the tibia |
| Tr-TiII | anterodorsal at the face of the trochanter | ventral at the proximal margin of the tibia |
| Fe-TaI | posterodorsal at the face of the femur | ventral at the proximal margin of the tarsus |
| Fe-TeI | dorsal at the face of the femur | tendon |
| Ti-TeI | dorsal at the face of the tibia | tendon |

**App. Table 9: In this study described extrinsic and intrinsic leg muscles in *Polyxenus lagurus.***

| *Polyxenus lagurus* | | |
| --- | --- | --- |
| Name of muscle | Insertion point 1 | Insertion point 2 |
| Ii-CoI | medial at the anterior intersegmental ventral insertion area | Anterior at the costa coxalis |
| Ii-CoII | mediolateral at the anterior intersegmental ventral insertion area | ventral/medial at the proximal margin of the coxa |
| Ii-CoIII | mediolateral at the posterior intersegmental ventral insertion area | Anterior at the costa coxalis |
| Ii-CoIV | mediolateral at the posterior intersegmental ventral insertion area | medial at the coxa costalis |
| Ii-PrI | mediolateral at the posterior intersegmental ventral insertion area | posteroventral at the proximal margin of the prefemur |
| Ii-PrII | medial at the anterior intersegmental ventral insertion area | anterior at the proximal margin of the prefemur slightly ventral of the contact point |
| Ii-FeI | medial at the posterior intersegmental ventral insertion area | anteroventral at the proximal margin of the femur |
| Ii-FeII | mediolateral at the posterior intersegmental ventral insertion area | posteroventral at the proximal margin of the femur |
| Mn-CoI | Muscle node located dorsally at the base of the coxa | posteroventral/-medial at the proximal margin of the coxa |
| Mn-FeI | Muscle node located dorsally at the base of the coxa | posterior at the proximal margin of the femur |
| Mn-FeII | Muscle node located dorsally at the base of the coxa | anterior at the face of the femur |
| Co-TrI | anteroventral/medial at the face of the coxa | anteroventral at the proximal margin of the trochanter |
| Co-PrI | ventral/medial at the proximal margin of the coxa | ventral at the proximal margin of the prefemur |
| Co-FeI | anterior at the costa coxalis | anterior at the proximal margin of the femur slightly dorsal of the contact point |
| Co-FeII | anteroventral/-medial at the costa coxalis | anteroventral at the proximal margin of the femur |
| Co-FeIII | anteroproximal at the costa coxalis | anterior at the face of the femur |
| Co-FeIV | anteroproximoventral at the face of the coxa | ventral at the proximal margin of the femur |
| Co-PoI | anteroventral/-medial at the costa coxalis | ventral at the proximal margin of the postfemur |
| Co-PoII | anterior at the costa coxalis | posterodorsal at the proximal margin of the postfemur |
| Co-TiI | anteroventral/-medial at the costa coxalis | anteroventral at the proximal margin of the tibia |
| Pr-FeI | anteroproximal at the face of the prefemur slightly ventral of the contact point | anterior at the proximal margin of the femur slightly ventral of the contact point |
| Fe-PoI | anteroproximal at the face of the femur | anterior at the proximal margin of the postfemur |
| Fe-PoII | anteroproximoventral at the face of the femur | anterior at the proximal margin of the postfemur |
| Fe-TiI | posteroproximoventral at the face of the femur | ventral at the proximal margin of the tibia |
| Fe-TiII | anterodorsal at the face of the femur | anterior at the posterior margin of the tibia |
| Fe-Ta2I | posteroproximal at the face of the femur | ventral at the proximal margin of the tarsus 2 |
| Fe-Ta2II | dorsal at the face of the femur | ventral at the proximal margin of the tarsus 2 |
| Fe-TeI | posteroproximodorsal at the face of the femur | tendon |
| Po-Ta2I | anteroproximodorsal at the face of the postfemur | ventral at the proximal margin of the tarsus 2 |
| Po-TeI | proximodorsal at the face of the postfemur | tendon |
| Ti-TeI | anterior and posterior at the face of the tibia | tendon |

**App. Table 10: In this study described extrinsic and intrinsic leg muscles in *Benoitolus siamensis.***

| *Benoitolus siamensis* | | |
| --- | --- | --- |
| Name of muscle | Insertion point 1 | Insertion point 2 |
| Ap-CoI | wide from medioventral to the middle between the lateral border of the bow-shaped apodeme and its middle | anterior at the proximal margin of the coxa |
| Ap-CoII | medioventral at the bow-shaped apodeme | posterior at the proximal margin of the coxa |
| Ap-CoIII | wide from medial to lateral of the bow-shaped apodeme and its middle | posterior at the proximal margin of the coxa |
| Ap-CoIV.I | tip of the spiculiform apodeme | anteromedian at the margin of the coxa |
| Ap-CoIV.II | tip of the spiculiform apodeme | medial at the sclerite ring |
| Co-PrI | anterior face of the coxa | dorsal at the proximal margin of the prefemur |
| Co-PrII | wide from median to posterior of the face of the coxa | ventral at the proximal margin of the coxa (process of the prefemur) |
| Pr-FeI | wide from anteroproximal to anterior of the face of the prefemur | dorsal at the proximal margin of the femur |
| Pr-FeII | wide from anteroproximal to anterior of the face of the prefemur | ventral at the proximal margin of the femur |
| Pr-FeIII | anterior of the face of the prefemur | ventral at the proximal margin of the femur |
| Pr-FeIV | posterodistal of the face of the prefemur | posterior at the proximal margin of the femur |
| Fe-PoI | anterodorsal at the face of the femur | Wide from anterior to ventral at the proximal margin of the postfemur |
| Fe-PoII | anteroproximal at the face of the femur | ventral at the proximal margin of the postfemur |
| Fe-PoIII | Wide from posterior to posteroproximal at the face of the femur | ventral at the proximal margin of the postfemur |
| Fe-TiI | dorsoanterior at the face of the femur | ventral at the proximal margin of the tibia |
| Fe-TiII | dorsoposterior at the face of the femur | ventral at the proximal margin of the tibia |
| Po-TiI | dorsoanterior at the face of the postfemur | ventroanterior at the proximal margin of the tibia |
| Po-TiII | dorsoposterior at the face of the postfemur | ventroposterior at the proximal margin of the tibia |
| Po-TaI | dorsoanterior at the face of the postfemur | ventral at the proximal margin of the tarsus |
| Po-TaII | dorsoposterior at the face of the postfemur | ventral at the proximal margin of the tarsus |
| Ti-TeI | two big attachment points: anterodorsal and posterodorsal at the face of the cuticle | tendon |
